# Machine Learning Models for Blood Glucose Level Prediction in Patients With Diabetes Mellitus: Systematic Review and Network Meta-Analysis

**DOI:** 10.2196/47833

**Published:** 2023-11-20

**Authors:** Kui Liu, Linyi Li, Yifei Ma, Jun Jiang, Zhenhua Liu, Zichen Ye, Shuang Liu, Chen Pu, Changsheng Chen, Yi Wan

**Affiliations:** 1 Department of Health Service Air Force Medical University Xi'an, Shaanxi China; 2 Department of Health Statistics Air Force Medical University Xi'an Shaanxi China

**Keywords:** machine learning, diabetes, hypoglycemia, blood glucose, blood glucose management

## Abstract

**Background:**

Machine learning (ML) models provide more choices to patients with diabetes mellitus (DM) to more properly manage blood glucose (BG) levels. However, because of numerous types of ML algorithms, choosing an appropriate model is vitally important.

**Objective:**

In a systematic review and network meta-analysis, this study aimed to comprehensively assess the performance of ML models in predicting BG levels. In addition, we assessed ML models used to detect and predict adverse BG (hypoglycemia) events by calculating pooled estimates of sensitivity and specificity.

**Methods:**

PubMed, Embase, Web of Science, and Institute of Electrical and Electronics Engineers Explore databases were systematically searched for studies on predicting BG levels and predicting or detecting adverse BG events using ML models, from inception to November 2022. Studies that assessed the performance of different ML models in predicting or detecting BG levels or adverse BG events of patients with DM were included. Studies with no derivation or performance metrics of ML models were excluded. The Quality Assessment of Diagnostic Accuracy Studies tool was applied to assess the quality of included studies. Primary outcomes were the relative ranking of ML models for predicting BG levels in different prediction horizons (PHs) and pooled estimates of the sensitivity and specificity of ML models in detecting or predicting adverse BG events.

**Results:**

In total, 46 eligible studies were included for meta-analysis. Regarding ML models for predicting BG levels, the means of the absolute root mean square error (RMSE) in a PH of 15, 30, 45, and 60 minutes were 18.88 (SD 19.71), 21.40 (SD 12.56), 21.27 (SD 5.17), and 30.01 (SD 7.23) mg/dL, respectively. The neural network model (NNM) showed the highest relative performance in different PHs. Furthermore, the pooled estimates of the positive likelihood ratio and the negative likelihood ratio of ML models were 8.3 (95% CI 5.7-12.0) and 0.31 (95% CI 0.22-0.44), respectively, for predicting hypoglycemia and 2.4 (95% CI 1.6-3.7) and 0.37 (95% CI 0.29-0.46), respectively, for detecting hypoglycemia.

**Conclusions:**

Statistically significant high heterogeneity was detected in all subgroups, with different sources of heterogeneity. For predicting precise BG levels, the RMSE increases with a rise in the PH, and the NNM shows the highest relative performance among all the ML models. Meanwhile, current ML models have sufficient ability to predict adverse BG events, while their ability to detect adverse BG events needs to be enhanced.

**Trial Registration:**

PROSPERO CRD42022375250; https://www.crd.york.ac.uk/prospero/display_record.php?RecordID=375250

## Introduction

Diabetes mellitus (DM) has become one of the most serious health problems worldwide [1], with more than 463 million (9.3%) patients in 2019; this number is predicted to reach 700 million (10.9%) in 2045 [2], which has resulted in growing concerns about the negative impacts on patients’ lives and the increasing burden on the health care system [3]. Furthermore, previous studies have shown that without appropriate medical care, DM can lead to multiple long-term complications in blood vessels, eyes, kidneys, feet (ulcers), and nerves [4-7]. Adverse blood glucose (BG) events are one of the most common short-term complications, including hypoglycemia with BG<70 mg/dL and hyperglycemia with BG>180 mg/dL. Hyperglycemia in patients with DM may lead to lower limb occlusions and extremity nerve damage, further leading to decay, necrosis, and local or whole-foot gangrene, even requiring amputation [8,9]. Hypoglycemia can cause serious symptoms, including anxiety, palpitation, and confusion in a mild scenario and seizures, coma, and even death in a severe scenario [10,11]. Thus, there is an imminent need for preventing adverse BG events.

Machine learning (ML) models use statistical techniques to provide computers with the ability to complete assignments by training themselves without being explicitly programmed [12]. However, ML models for managing BG requires huge amounts of BG data, which cannot be satisfied by the multiple data points generated by the traditional finger-stick glucose meter [13]. With the introduction of the continuous glucose monitoring (CGM) device, which typically produces a BG reading every 5 minutes all day long, the size of the data set of BG readings is sufficient to be used in ML models [14].

Recently, there has been an immense surge in using ML technologies for predicting DM complications. Regarding BG management, previous studies have developed different types of ML models, including random forest (RF) models, support vector machines (SVMs), neural network models (NNMs), and autoregression models (ARMs), using CGM data, electronic health records (EHRs), electrocardiograph (ECG), electroencephalograph (EEG), and other information (ie, biochemical indicators, insulin intake, exercise, and meals) [10,15-20]. However, the performance of different models in these studies was not quite consistent. For instance, in terms of BG level prediction, Prendin et al [21] showed that the SVM achieved a lower root mean square error (RMSE) than the ARM, while Zhu et al [22] showed a different result.

Therefore, this meta-analysis aimed to comprehensively assess the performance of ML models in BG management in patients with DM.

## Methods

### Search Strategy and Study Selection

The study protocol has been registered in the international prospective register of systematic reviews (PROSPERO; registration ID: CRD42022375250). Studies on BG levels or adverse BG event prediction or detection using ML models were eligible, with no restrictions on language, investigation design, or publication status. PubMed, Embase, Web of Science, and Institute of Electrical and Electronics Engineers (IEEE) Explore databases were systematically searched from inception to November 2022. Keywords used for study repository searches were (“machine learning” OR “artificial intelligence” OR “logistic model” OR “support vector machine” OR “decision tree” OR “cluster analysis” OR “deep learning” OR “random forest”) AND (“hypoglycemia” OR “hyperglycemia” OR “adverse glycemic events”) AND (“prediction” OR “detection”). Details regarding the search strategies are summarized in Multimedia Appendix 1. Manual searches were added to review reference lists in relevant studies.

### Selection Criteria

Inclusion criteria were as follows: (1) participants in the studies were diagnosed with DM; (2) study endpoints were hypoglycemia, hyperglycemia, or BG levels; (3) the studies established at least 2 or more types of ML models for prediction of BG levels and 1 or more types of ML models for prediction or detection of adverse BG events; (4) the studies reported the performance of ML models with statistical or clinical metrics; (5) the studies contained the development and validation of ML models; and (6) study outcomes were means (SDs) of performance metrics of test data for prediction of BG levels and sensitivity and specificity of test data for prediction or detection of adverse BG events.

Exclusion criteria were as follows: (1) studies did not report on the derivation of ML models, (2) studies were based only on physiological or control-oriented ML models, (3) studies could not reproduce true positives, true positives, false negatives, and false positives for prediction or detection of adverse BG events, (4) studies were reviews, systematic reviews, animal studies, or irretrievable and repetitive papers, and (5) studies had unavailable full text or outcome metrics.

Authors KL and LYL screened and selected studies independently based on the criteria mentioned before. Authors KL and YM extracted and recorded the data from the selected studies. Conflicts were resolved by reaching a consensus. The study strictly followed the PRISMA (Preferred Reporting Items for Systematic Reviews and Meta-Analysis) statement (Multimedia Appendix 2) [23-25].

### Data Extraction and Management

Two reviewers independently carried out data extraction and quality assessment. If a single study included more than 1 extractable test results for the same ML model, the best result was extracted. If a single study included 2 or more models, the performance metrics of each model were extracted. For studies predicting BG levels, RMSEs based on different prediction horizons (PHs) were extracted. For studies predicting or detecting adverse BG events, the sensitivity, specificity, and precision of reproducing the 2×2 contingency table were extracted.

Specifically, the following information was extracted:

- General characteristics: first author, publication year, country, data source, and study purpose (ie, predicting or detecting hypoglycemia)
- Experimental information: participants (type of DM, type 1 or 2), sample size (patients, data points, and hypoglycemia), demographic information, models, study place and time, model parameters (ie, input and PHs), model performance metrics, threshold of BG levels for hypoglycemia, and reference (ie, finger-stick)

### Methodological Quality Assessment of Included Reviews

The Quality Assessment of Diagnostic Accuracy Studies (QUADAS-2) tool was applied to assess the quality of included studies based on patient selection (5 items), index test (3 items), reference standard (4 items), and flow and timing (4 items). All 4 domains were used for assessing the risk of bias, and the first 3 domains were used to assess the consensus of applicability. Each domain has 1 query in relation to the risk of bias or applicability consisting of 7 questions [26].

### Data Synthesis and Statistical Analysis

The performance metrics of ML models used to predict BG levels, predict adverse BG events, and detect adverse BG events were assessed independently. The performance metrics were the RMSE of ML models in predicting BG levels and the sensitivity and specificity of ML models in predicting or detecting adverse BG events. A network meta-analysis was conducted for BG level–based studies to assess the global and local inconsistency between studies and plotted the surface under the cumulative ranking (SUCRA) curve of every model to calculate relative ranks. For event-based studies, pooled sensitivity, specificity, the positive likelihood ratio (PLR), and the negative likelihood ratio (NLR) with 95% CIs were calculated. Study heterogeneity was assessed by calculating I² values based on multivariate random-effects meta-regression that considered within- and between-study correlation and classifying them into quartiles (0% to <25% for low, 25% to <50% for low-to-moderate, 50% to <75% for moderate-to-high, and >75% for high heterogeneity) [27,28]. Furthermore, meta-regression was used to evaluate the source of heterogeneity for both BG level–based and adverse event–based studies. The summary receiver operating characteristic (SROC) curve of every model was also used to evaluate the overall sensitivity and specificity. Publication bias was assessed using the Deek funnel plot asymmetry test.

Furthermore, BG level–based studies were divided into 4 subgroups based on different PHs (15, 30, 45, 60 minutes), and adverse event–based studies were analyzed using different types of models (ie, NNM, RF, and SVM). A 2-sided *P* value of <.05 was considered statistically significant. All statistical analyses were performed using Stata 17 (Stata Corp) and Review Manager (RevMan; Cochrane) version 5.3.

## Results

### Search Results

**Figure 1:**
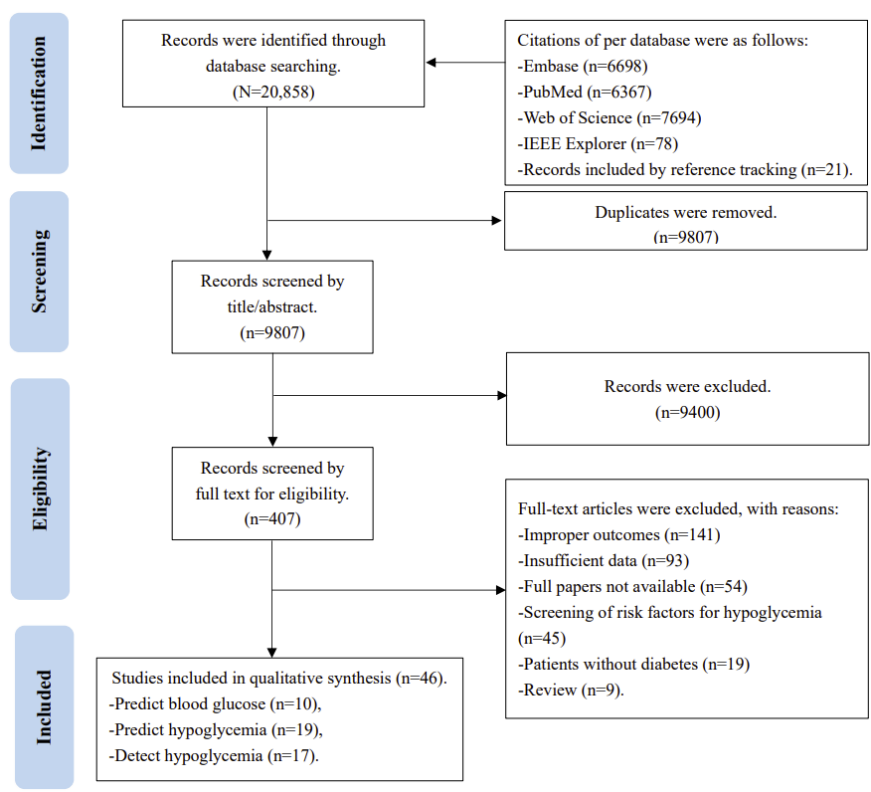
Flow diagram of identifying and including studies. IEEE: Institute of Electrical and Electronics Engineers.

A total of 20,837 studies were identified through systematically searching the predefined electronic databases; these also included 21 studies found using reference tracking [10,29-48]. Of the 20,837 studies, 9807 (47.06%) were retained after removing duplicates. After screening titles and abstracts, 9400 (95.85%) studies were excluded owing to reporting irrelevant topics or no predefined outcomes. The remaining 407 (4.15%) studies were retrieved for full-text evaluation. Of these, 361 (88.7%) studies were excluded for various reasons, and therefore 46 (11.3%) studies were included in the final meta-analysis (Figure 1).

### Description of Included Studies

As studies on hyperglycemia were insufficient for analysis, we selected studies on hypoglycemia to assess the ability of ML models to predict adverse BG events. In total, the 46 studies included 28,775 participants: n=428（1.49%）for predicting BG levels, n=28,138 (97.79%) for predicting adverse BG events, and n=209 (0.72%) for detecting adverse BG events. Of the 46 studies, 10 (21.7%) [20-22,49-55] predicted BG levels (Table 1), 19 (41.3%) [15,29-39,47,48,56-60] predicted adverse BG events (Table 2), and the remaining 17 (37%) [10,16,40-46,61-68] detected adverse BG events (Table 3).

**Table 1:** Baseline characteristics of BG^a^ level-based studies (N=10).

First author (year), country	Data source	Sample size			Demographic information		Object; setting		Model; PH^b^ (minutes); input		Performance metrics
		Patients, n	Data points, n								
Pérez-Gandía (2010), Spain [20]	CGM^c^ device	15	728	—^d^		T1DM^e^; out		Models: NNM^f^, ARM^g^ PH: 15, 30 Input: CGM data		RMSE^h^, delay	
Prendin (2021) United States [21]	CGM device	Real (n=141)	350,000	Age		T1DM; out		ARM, autoregressive moving average (ARMA), autoregressive integrated moving average (ARIMA), SVM^i^, RF^j^ feed-forward neural network (fNN), long short-term memory (LSTM) PH: 30 Input: CGM data		RMSE, coefficient of determination (COD) sensibility, delay, precision *F*_1_ score, time gain	
Zhu (2020) England [22]	Ohio T1DM, UVA/Padova T1D	Real (n=6), simulated (n=10)	1,036,800	—		T1DM; out		DRNN^k^, NNM, SVM, ARM PH:30 Input: BG level, meals, exercise, meal times		RMSE, mean absolute relative difference (MARD) time gain	
D'Antoni (2020), Italy [49]	Ohio T1DM	6	—	Age, sex ratio		T1DM; out		ARJNN^l^, RF, SVM, autoregression (AR), one symbolic model (SAX), recurrent neural network (RNN), one neural network model (NARX), jump neural network (JNN), delayed feed-forward neural network model (DFFNN) PH: 15, 30 Input: CGM data		RMSE	
Amar (2020), Israel [50]	CGM device, insulin pump	141	1,592,506	Age, sex ratio, weight, BMI, duration of DM		T1DM; in		ARM, gradually connected neural network (GCN), fully connected (FC [neural network]), light gradient boosting machine (LCBM), RF PH: 30, 60 Input: CGM data		RMSE, Clarke error grid (CEG)	
Li (2020), England [51]	UVA/Padova T1D	Simulated (n=10)	51,840	—		T1DM; out		GluNet, NNM, SVM, latent variable with exogenous input (LVX), ARM PH: 30, 60 Input: BG level, meals, exercise		RMSE, MARD, time lag	
Zecchin (2012), Italy [52]	UVA/Padova T1D, CGM device	Simulated (n=20), real (n=15)	—	—		T1DM; out		Neural network–linear prediction algorithm (NN-LPA), NN, ARM PH: 30 Input: meals, insulin		RMSE, energy of second-order differences (ESOD), time gain, J index	
Mohebbi (2020), Denmark [53]	Cornerstones4Care platform	Real (n=50	—	—		T1DM; in		LSTM, ARIMA PH: 15, 30, 45, 60, 90		RMSE, MAE	
Daniels (2022), England [54]	CGM device	Real (n=12)	—	Sex ratio		T1DM; out		Convolutional recurrent neural network (CRNN), SVM PH: 30, 45, 60, 90, 120 Input: BG level, insulin, meals, exercise		RMSE, MAE, CEG, time gain	
Alfian (2020), Korea [55]	CGM device	Real (n=12)	26,723	—		—		SVM, k-nearest neighbor k-nearest neighbor (kNN), DT^m^, RF, AdaBoost, XGBoost^n^, NNM PH: 15, 30 Input: CGM data		RMSE, glucose-specific root mean square error (gRMSE), R2 score, mean absolute percentage error (MAPE)	

^a^BG: blood glucose.

^b^PH: prediction horizon.

^c^CGM: continuous glucose monitoring.

^d^Not applicable.

^e^T1DM: type 1 diabetes mellitus.

^f^NNM: neural network model.

^g^ARM: autoregression model.

^h^RMSE: root mean square error.

^i^SVM: support vector machine.

^j^RF: random forest.

^k^DRNN: dilated recurrent neural network.

^l^ARJNN: ARTiDe jump neural network.

^m^DT: decision tree.

^n^XGBoost: Extreme Gradient Boosting.

**Table 2:** Baseline characteristics of studies predicting adverse BG^a^ events (N=19).

First author (year), country	Data source	Sample size			Object; setting	Model		Time		Age (years), mean (SD)/range		Threshold
		Patients, n	Data points, n	Hypoglycemia, n								
Pils (2014), United States [39]	CGM^b^ device	2	2518	152	T1DM^c^; out	SVM^d^	All		—^e^		3.9	
Seo (2019), Korea [15]	CGM device	104	7052	412	DM^f^; out	RF^g^, SVM, k-nearest neighbor (kNN), logistic regression (LR)	Postprandial		52		3.9	
Parcerisas (2022), Spain [29]	CGM device	10	67	22	T1DM; out	SVM	Nocturnal		31.8 (SD 16.8)		3.9	
Stuart (2017), Greece [30]	EHRs^h^	9584	—	1327	DM; in	Multivariable logistic regression (MLR)	All		—		4	
Bertachi (2020), Spain [31]	CGM device	10	124	39	T1DM; out	SVM	Nocturnal		31.8 (SD 16.8)		3.9	
Elhadd (2020), Qatar [32]	—	13	3918	172	T2DM; out	XGBoost^i^	All		35-63		—	
Mosquera-Lopez (2020), United States [33]	CGM device	10	117	17	T1DM; out	SVM	Nocturnal		33.7 (SD 5.8)		3.9	
Mosquera-Lopez (2020), United States [33]	CGM device	20	2706	258	T1DM; out	SVM	Nocturnal		—		3.9	
Ruan (2020), England [34]	EHRs	17,658	3276	703	T1DM; in	XGBoost, LR, stochastic gradient descent (SGD), kNN, DT^j^, SVM, quadratic discriminant analysis (QDA), RF, extra tree (ET), linear discriminant analysis (LDA), AdaBoost, bagging	All		66 (SD 18)		4	
Güemes (2020), United States [35]	CGM device	6	55	6	T1DM; out	SVM	Nocturnal		40-60		3.9	
Jensen (2020), Denmark [36]	CGM device	463	921	79	T1DM; out	LDA	Nocturnal		43 (SD 15)		3	
Oviedo (2019), Spain [37]	CGM device	10	1447	420	T1DM; out	SVM	Postprandial		41 (SD 10)		3.9	
Toffanin (2019), Italy [38]	CGM device	20	7096	36	T1DM; out	Individual model-based	All		46		3.9	
Bertachi (2018), United States [47]	CGM device	6	51	6	T1DM; out	NNM^k^	Nocturnal		40-60		3.9	
Eljil (2014), United Arab Emirates [48]	CGM device	10	667	100	T1DM; out	Bagging	All		25		3.3	
Dave (2021), United States [56]	CGM device	112	546,640	12,572	T1DM; out	RF	All		12.67 (SD 4.84)		3.9	
Marcus (2020), Israel [57]	CGM device	11	43,533	5264	T1DM; out	Kernel ridge regression (KRR)	All		18-39		3.9	
Reddy (2019), United States [58]	—	55	90	29	T1DM; out	RF	—		33 (SD 6)		3.9	
Sampath (2016), Australia [59]	—	34	150	40	T1DM; out	Ranking aggregation (RA)	Nocturanl		—		—	
Sudharsan (2015), United States [60]	—	—	839	428	T2DM; out	RF	All		—		3.9	

^a^BG: blood glucose.

^b^CGM: continuous glucose monitoring.

^c^T1DM: type 1 diabetes mellitus.

^d^SVM: support vector machine.

^e^Not applicable.

^f^DM: diabetes mellitus.

^g^RF: random forest.

^h^EHR: electronic health record.

^i^XGBoost: Extreme Gradient Boosting.

^j^DT: decision tree.

^k^NNM: neural network model.

**Table 3:** Baseline characteristics of studies detecting adverse BG^a^ events (N=17).

First author (year), country	Data source	Sample size			Object; setting	Model		Time		Age (years), mean (SD)/range		Threshold
		Patients, n	Data points, n	Hypoglycemia, n								
Jin (2019), United States [10]	EHRs^b^	—^c^	4104	132	T1DM^d^; in	Linear discriminant analysis (LDA)	All		—		—	
Nguyen (2013), Australia [16]	EEG^e^	5	144	76	T1DM; in	Levenberg-Marquardt (LM), genetic algorithm (GA)	All		12-18		3.3	
Chan (2011), Australia [40]	CGM^f^ device	16	100	52	T1DM; experimental	Feed-forward neural network (fNN)	Nocturnal		14.6 (SD 1.5)		3.3	
Nguyen (2010), Australia [41]	EEG	6	79	27	T1DM; experimental	Block-based neural network (BRNN)	Nocturnal		12-18		3.3	
Rubega (2020), Italy [42]	EEG	34	2516	1258	T1DM; experimental	NNM^g^	All		55 (SD 3)		3.9	
Chen (2019), United States [43]	EEG	—	300	11	DM^h^; in	Logistic regression (LR)	All		—		—	
Jensen (2013), Denmark [44]	CGM device	10	1267	160	T1DM; experimental	SVM^i^	All		44 (SD 15)		3.9	
Skladnev (2010), Australia [45]	CGM device	52	52	11	T1DM; in	fNN	Nocturnal		16.1 (SD 2.1)		3.9	
Iaione (2005), Brazil [46]	EEG	8	1990	995	T1DM; experimental	NNM	Morning		35 (SD 13.5)		3.3	
Nuryani (2012), Australia [61]	ECG	5	575	133	DM; in	SVM, linear multiple regression (LMR)	All		16 (SD 0.7)		3.0	
San (2013), Australia [62]	ECG	15	440	39	T1DM; in	Block-based neural network (BBNN), wavelet neural network (WNN), fNN, SVM	All		14.6 (SD 1.5)		3.3	
Ling (2012), Australia [63]	ECG	16	269	54	T1DM; in	Fuzzy reasoning model (FRM), fNN, multiple regression–fuzzy inference system (MR-FIS)	Nocturnal		14.6 (SD 1.5)		3.3	
Ling (2016), Australia [64]	ECG	16	269	54	T1DM; in	Extreme learning machine–based neural network (ELM-NN), particle swarm optimization–based neural network (PSO-NN), MR-FIS, LMR, fuzzy inference system (FIS)	Nocturnal		14.6 (SD 1.5)		3.3	
Nguyen (2012), Australia [65]	EEG	5	44	20	T1DM; in	NNM	—		12-18		3.3	
Ngo (2020), Australia [66]	EEG	8	135	53	T1DM; in	BRNN	Nocturnal		12-18		3.9	
Ngo (2018), Australia [67]	EEG	8	54	26	T1DM; in	BRNN	Nocturnal		12-18		3.9	
Nuryani (2010), Australia [68]	ECG	5	27	8	T1DM; experimental	Fuzzy support vector machine (FSVM), SVM	Nocturnal		16 (SD 0.7)		3.3	

^a^BG: blood glucose.

^b^EHR: electronic health record.

^c^Not applicable.

^d^T1DM: type 1 diabetes mellitus.

^e^EEG: electroencephalograph.

^f^CGM: continuous glucose monitoring.

^g^NNM: neural network model.

^h^DM: diabetes mellitus.

^i^SVM: support vector machine.

As shown in Tables 1-3, 40 (87%) studies [10,16,20-22,29,31,33-42,44-59,62-68] included participants with type 1 diabetes mellitus (T1DM), 2 (4.3%) studies [32,60] included participants with type 2 diabetes mellitus (T2DM), and the remaining 4 (8.7%) studies [15,30,43,61] did not specify the type of DM. Regarding the data source of ML models, CGM devices were involved in 22 (47.8%) studies [15,20,21,29,31,33,35-40,44,45,47,48,50,52,54-57], EEG signals were used in 8 (17.4%) studies [16,41-43,46,65-67], ECG signals were involved in 5 (10.9%) studies [61-64,68], EHRs were used in 3 (6.5%) studies [10,30,34], data generated by the UVA/Padova T1D simulator were used in 3 (6.5%) studies [22,51,52], the Ohio T1DM data set was used in 2 (4.3%) studies [22,49], and 4 (8.7%) studies [32,58-60] did not report the source of data. Regarding the setting of data collection, 24 (52.2%) studies [15,20-22,29,31-33,35-39,47-49,51,52,54,56-60] were conducted in an out-of-hospital setting, 13 (28.3%) studies [10,16,34,43,50,53,61-67] were conducted in an in-hospital setting, 6 (13%) studies [40-42,44,46,68] were conducted in an experimental setting, and the remaining 1 (2.2%) study [55] did not specify the environment. Regarding when adverse BG events occurred in the 36 (78.3%) adverse event–based studies, 15 (41.7%) [29,31,33,35,36,40,41,45,47,59,63,64,66-68] reported nocturnal hypoglycemia, 16 (44.4%) [10,16,30,32,34,38,39,42-44,48,56,57,60-62] were not specific about the time of day, 2 (5.6%) [15,37] reported postprandial hypoglycemia, 1 (2.8%) [46] reported morning hypoglycemia, and the remaining 2 (5.6%) [58,65] did not report the time setting. To carry out the network meta-analysis of BG level–based studies, we chose the RMSE as the outcome to be compared.

### Quality Assessment of Included Studies

**Figure 2:**
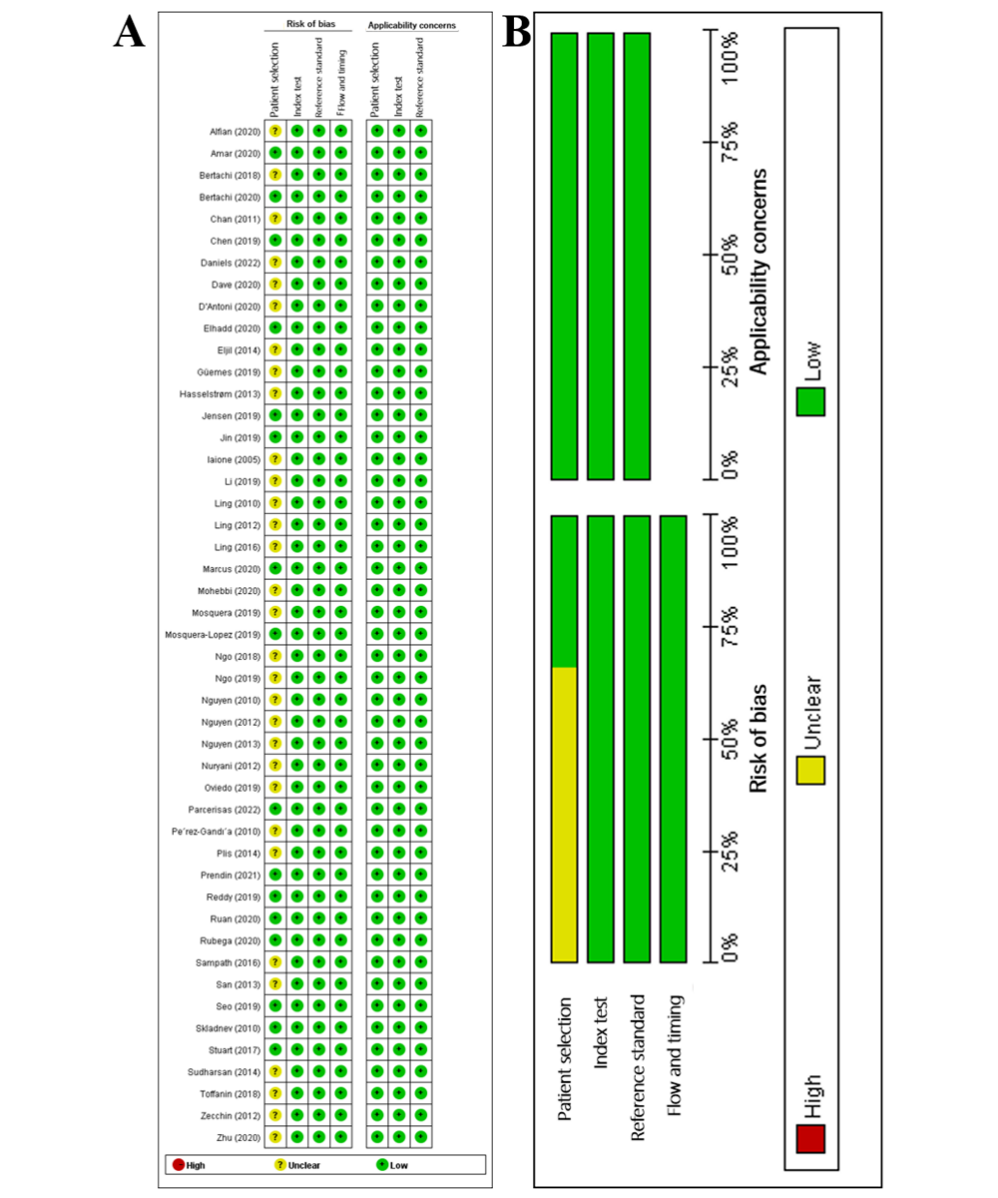
Quality assessment of included studies. Risk of bias and applicability concerns graph (A) and risk of bias and applicability concerns summary (B).

The quality assessment results using the QUADAS-2 tool showed that more than half of all included studies did not report the patient selection criteria in detail, which led to low-quality patient selection (Figure 2). Furthermore, the diagnosis of hypoglycemia using blood or the CGM device was considered high quality in the reference test in our study.

### Statistical Analysis

#### Machine Learning Models for Predicting Blood Glucose Levels

**Figure 3:**
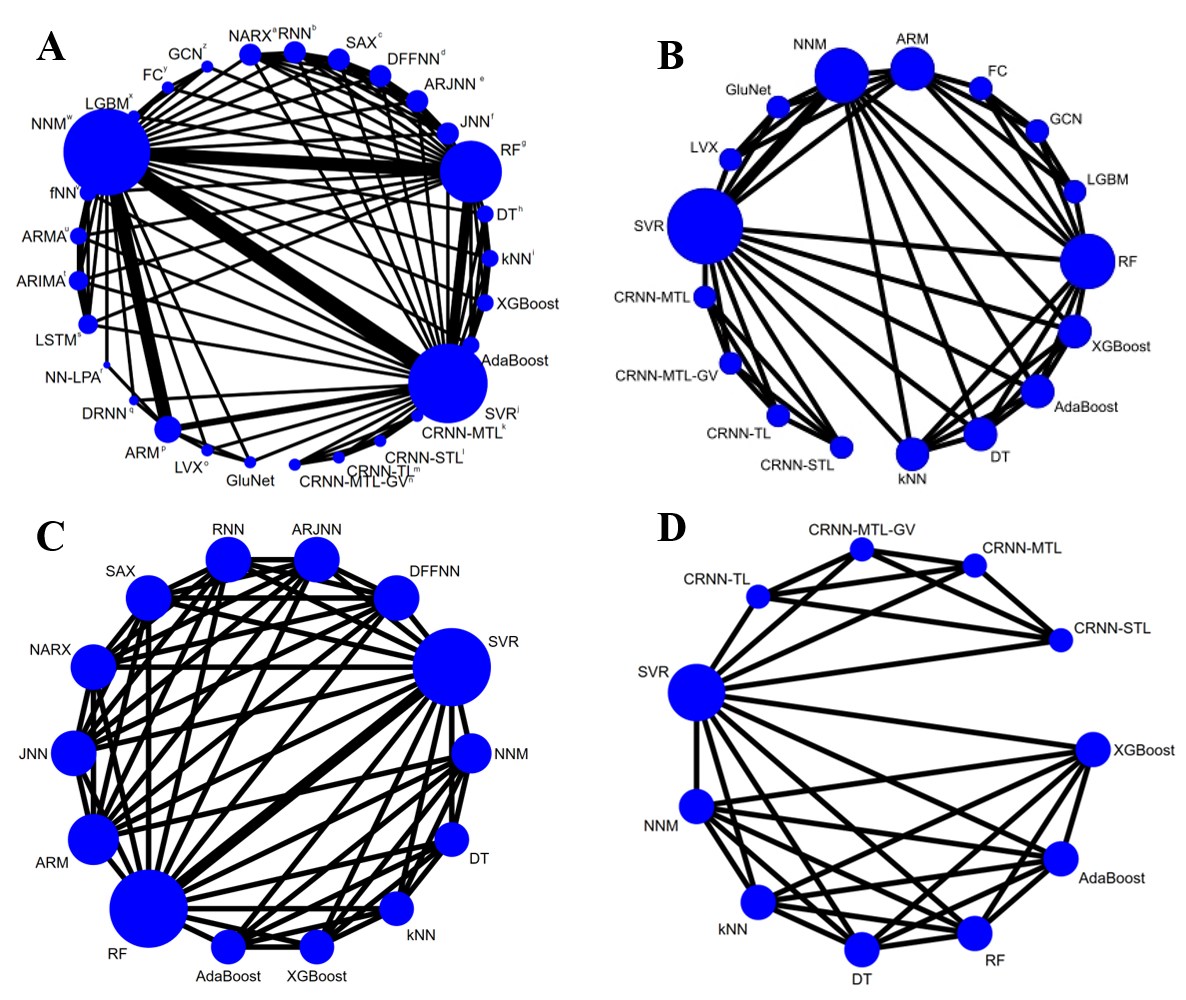
Network map of ML models for predicting BG levels in different PHs. PH=30 (A), 60 (B), 15 (C), and 45 minutes (D). ARIMA: autoregressive integrated moving average; ARM: autoregression model; ARMA: autoregressive moving average; ARJNN: ARTiDe jump neural network; BG: blood glucose; CRNN-MTL: convolutional recurrent neural network multitask learning; CRNN-MTL-GV: convolutional recurrent neural network multitask learning glycemic variability; CRNN-STL: convolutional recurrent neural network single-task learning; CRNN-TL: convolutional recurrent neural network transfer learning; DFFNN: delayed feed-forward neural network; DRNN: dilated recurrent neural network; DT: decision tree; FC: fully connected (neural network); fNN: feed-forward neural network; GCN: gradually connected neural network; JNN: jump neural network; kNN: k-nearest neighbor; LGBM: light gradient boosting machine; LSTM: long short-term memory; LVX: latent variable with exogenous input; ML: machine learning; NARX: one neural network model; NN-LPA: neural network–linear prediction algorithm; NNM: neural network model; PH: prediction horizon; RF: random forest; RNN: recurrent neural network; SAX: one symbolic model; SVR: support vector regression.

**Figure 4:**
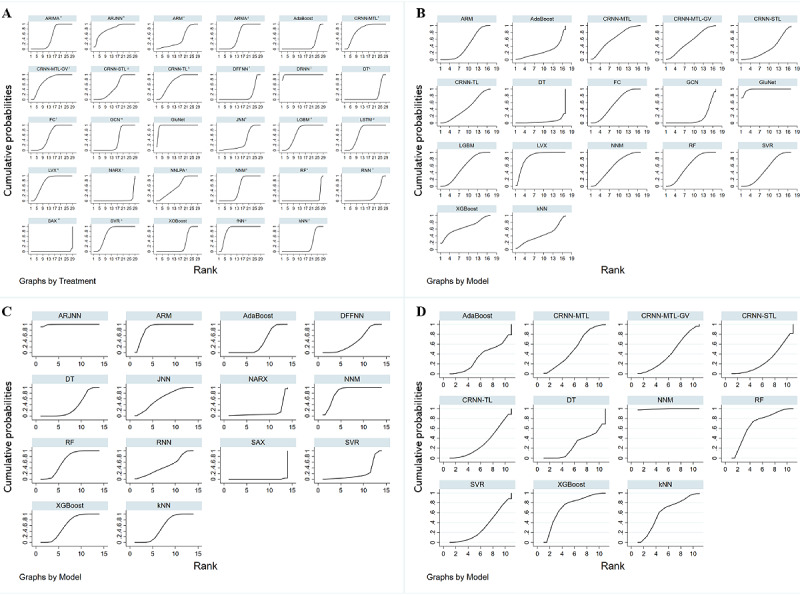
SUCRA curves of ML models for predicting BG levels in different PHs. PH=30 (A), 60 (B), 15 (C), and 45 minutes (D). ARIMA: autoregressive integrated moving-average; ARM: autoregression model; ARMA: autoregressive moving average; ARJNN: ARTiDe jump neural network; BG: blood glucose; CRNN-MTL: convolutional recurrent neural networks multitask learning; CRNN-MTL-GV: convolutional recurrent neural networks multitask learning glycemic variability; CRNN-STL: convolutional recurrent neural networks single-task learning; CRNN-TL: convolutional recurrent neural networks transfer learning; DFFNN: delayed feed-forward neural network; DRNN: dilated recurrent neural network; DT: decision tree; FC: fully connected (neural network); fNN: feed-forward neural network; GCN: gradually connected neural network; JNN: jump neural network; kNN: k-nearest neighbor; LGBM: light gradient boosting machine; LSTM: long short-term memory; LVX: latent variable with exogenous input; ML: machine learning; NARX: one neural network model; NN-LPA: neural network–linear prediction algorithm; NNM: neural network model; PH: prediction horizon; RF: random forest; RNN: recurrent neural network; SAX: one symbolic model; SVR: support vector regression.

Network meta-analysis was conducted to evaluate the performance of different ML models. For PH=30 minutes, 10 (21.7%) studies [20-22,49-55] with 32 different ML models were included, and the network map is shown in Figure 3A. The mean RMSE was 21.40 (SD 12.56) mg/dL. Statistically significant inconsistency was detected using the inconsistency test(^2^=87.11, *P*<.001), as shown in the forest plot in Multimedia Appendix 1. Meta-regression indicated that I² for the RMSE was 60.75%, and the source of heterogeneity analysis showed that place and validation type were statistically significant (*P*<.001). The maximum SUCRA value was 99.1 for the dilated recurrent neural network (DRNN) model with a mean RMSE of 7.80 (SD 0.60) mg/dL [22], whereas the minimum SUCRA value was 0.4 for 1 symbolic model with a mean RMSE of 71.4 (SD 21.9) mg/dL [49]. The relative ranks of the ML models are shown in Table 4, and the SUCRA curves are shown in Figure 4A. Publication bias was tested using the Egger test (*P*=.503), indicating no significant publication bias.

For PH=60 minutes, 4 (8.7%) studies [50,51,55] with 17 different ML models were included, and the network map is shown in Figure 3B. The mean RMSE was 30.01 (SD 7.23) mg/dL. Statistically significant inconsistency was detected using the inconsistency test (^2^=8.82, *P*=.012), as shown in the forest plot in Multimedia Appendix 3. Meta-regression indicated that none of the sample size, reference, place, validation type, and model type was a source of heterogeneity. The maximum SUCRA value was 97.8 for the GluNet model with a mean RMSE of 19.90 (SD 3.17) mg/dL [51], while the minimum SUCRA value was 4.5 for the decision tree (DT) model with a mean RMSE of 32.86 (SD 8.81) mg/dL [55]. The relative ranks of the ML models are shown in Table 5, and the SUCRA curves are shown in Figure 4B. No significant publication bias was detected using the Egger test (*P*=.626).

For PH=15 minutes, 3 (6.5%) studies [20,49,55] with 14 different ML models were included, and the network map is shown in Figure 3C. The mean RMSE was 18.88 (SD 19.71) mg/dL. Statistically significant inconsistency was detected using the inconsistency test (^2^=28.29, *P*<.001), as shown in the forest plot in Multimedia Appendix 4. Meta-regression showed that I² was 41.28%, and the model type and sample size both were the source of heterogeneity, with *P*=.002 and .037, respectively. The maximum SUCRA value was 99.1 for the ARTiDe jump neural network (ARJNN) model with a mean RMSE of 9.50 (SD 1.90) mg/dL [49], while the minimum SUCRA value was 0.3 for the SVM with a mean RMSE of 13.13 (SD 17.30) mg/dL [55]. The relative ranks of the ML models are shown in Table 6, and SUCRA curves are shown in Figure 4C. Statistically significant publication bias was detected using the Egger test (*P*=.003).

For PH=45 minutes, only 2 (4.3%) studies [54,55] with 11 different ML models were included, and the network map is shown in Figure 3D. The mean RMSE was 21.27 (SD 5.17) mg/dL. Statistically significant inconsistency was detected using the inconsistency test (^2^=6.92, *P*=.009), as shown in the forest plot in Multimedia Appendix 5. Meta-regression indicated significant heterogeneity from the model type (*P*=.006). The maximum SUCRA value was 99.4 for the NNM with a mean RMSE of 10.65 (SD 3.87) mg/dL [55], while the minimum SUCRA value was 26.3 for the DT model with a mean RMSE of 23.35 (6.36) mg/dL [55]. The relative ranks of the ML models are shown in Table 7, and SUCRA curves are shown in Figure 4D. Statistically significant publication bias was detected using the Egger test (*P*<.001).

**Table 4:** Relative ranks of ML^a^ models for predicting BG^b^ levels in PH^c^=30 minutes.

ML model	SUCRA^d^	Relative rank
NNM^e^	52.0	14.4
ARM^f^	39.6	17.9
ARJNN^g^	79.5	6.8
RF^h^	6.9	27.1
SVM^i^	73.3	8.5
One symbolic model (SAX)	0.4	28.9
Recurrent neural network (RNN)	19.0	23.7
One neural network model (NARX)	3.9	27.9
Jump neural network (JNN)	36.0	18.9
Delayed feed-forward neural network model (DFFNN)	15.8	24.6
Gradually connected neural network (GCN)	41.1	17.5
Fully connected (FC [neural network])	58.1	12.7
Light gradient boosting machine (LGBM)	69.3	9.6
DRNN^j^	99.1	1.2
Autoregressive moving average (ARMA)	54.3	13.8
Autoregressive integrated moving average (ARIMA)	46.6	16.0
Feed-forward neural network (fNN)	86.3	4.8
Long short-term memory (LSTM)	69.1	9.7
GluNet	96.4	2.0
Latent variable with exogenous input (LVX)	75.2	7.9
Neural network–linear prediction algorithm (NN-LPA)	60.0	12.2
Convolutional recurrent neural network multitask learning (CRNN-MTL)	77.5	7.3
Convolutional recurrent neural network multitask learning glycemic variability (CRNN-MTL-GV)	77.2	7.4
Convolutional recurrent neural network transfer learning (CRNN-TL)	71.8	8.9
Convolutional recurrent neural network single-task learning (CRNN-STL)	52.0	14.4
k-Nearest neighbor (kNN)	26.0	21.7
DT^k^	16.2	24.5
AdaBoost	18.0	24.0
XGBoost^l^	29.2	20.8

^a^ML: machine learning.

^b^BG: blood glucose.

^c^PH: prediction horizon.

^d^SUCRA: surface under the cumulative ranking.

^e^NNM: neural network model.

^f^ARM: autoregression model.

^g^ARJNN: ARTiDe jump neural network.

^h^RF: random forest.

^i^SVM: support vector machine.

^j^DRNN: dilated recurrent neural network.

^k^DT: decision tree.

^l^XGBoost: Extreme Gradient Boosting.

**Table 5:** Relative ranks of ML^a^ models for predicting BG^b^ levels in PH^c^=60 minutes.

ML model	SUCRA^d^	Relative rank
ARM^e^	41.0	10.4
Gradually connected neural network (GCN)	14.2	14.7
Fully connected (FC [neural network])	55.7	8.1
Light gradient boosting machine (LGBM)	56.0	8.0
RF^f^	59.7	7.5
GluNet	97.8	1.4
NNM^g^	59.9	7.4
SVM^h^	49.5	9.1
Latent variable with exogenous input (LVX)	85.9	3.3
Convolutional recurrent neural network multitask learning (CRNN-MTL)	61.4	7.2
Convolutional recurrent neural network multitask learning glycemic variability (CRNN-MTL-GV)	54.2	8.3
Convolutional recurrent neural network transfer learning (CRNN-TL)	44.5	9.9
Convolutional recurrent neural network single-task learning (CRNN-STL)	32.5	11.8
k-Nearest neighbor (kNN)	42.5	10.2
DT^i^	4.5	16.3
AdaBoost	24.1	13.1
XGBoost^j^	66.5	6.4

^a^ML: machine learning.

^b^BG: blood glucose.

^c^PH: prediction horizon.

^d^SUCRA: surface under the cumulative ranking.

^e^ARM: autoregression model.

^f^RF: random forest.

^g^NNM: neural network model.

^h^SVM: support vector machine.

^i^DT: decision tree.

^j^XGBoost: Extreme Gradient Boosting.

**Table 6:** Relative ranks of ML^a^ models for predicting BG^b^ levels in PH^c^=15 minutes.

ML model	SUCRA^d^	Relative rank
NNM^e^	84.4	3.0
ARM^f^	86.8	2.7
ARJNN^g^	99.1	1.1
RF^h^	64.6	5.6
SVM^i^	20.9	11.3
One symbolic model (SAX)	0.3	14.0
Recurrent neural network (RNN)	45.9	8.0
One neural network model (NARX)	11.8	12.5
Jump neural network (JNN)	62.2	5.9
Delayed feed-forward neural network model (DFFNN)	39.6	8.9
k-Nearest neighbor (kNN)	53.7	7.0
DT^j^	33.3	9.7
AdaBoost	36.8	9.2
XGBoost^k^	60.8	6.1

^a^ML: machine learning.

^b^BG: blood glucose.

^c^PH: prediction horizon.

^d^SUCRA: surface under the cumulative ranking.

^e^NNM: neural network model.

^f^ARM: autoregression model.

^g^ARJNN: ARTiDe jump neural network.

^h^RF: random forest.

^i^SVM: support vector machine.

^j^DT: decision tree.

^k^XGBoost: Extreme Gradient Boosting.

**Table 7:** Relative ranks of ML^a^ models for predicting BG^b^ levels in PH^c^=45 minutes.

ML model	SUCRA^d^	Relative rank
Convolutional recurrent neural network multitask learning (CRNN-MTL)	52.1	5.8
Convolutional recurrent neural network multitask learning glycemic variability (CRNN-MTL-GV)	41.8	6.8
Convolutional recurrent neural network transfer learning (CRNN-TL)	31.6	7.8
Convolutional recurrent neural network single-task learning (CRNN-STL)	27.5	8.2
SVM^e^	32.0	7.8
k-Nearest neighbor (kNN)	61.4	4.9
DT^f^	26.3	8.4
RF^g^	70.3	4.0
AdaBoost	34.1	7.6
XGBoost^h^	73.5	3.7
NNM^i^	99.4	1.1

^a^ML: machine learning.

^b^BG: blood glucose.

^c^PH: prediction horizon.

^d^SUCRA: surface under the cumulative ranking.

^e^SVM: support vector machine.

^f^DT: decision tree.

^g^RF: random forest.

^h^XGBoost: Extreme Gradient Boosting.

^i^NNM: neural network model.

#### Machine Learning Models for Predicting Hypoglycemia

**Figure 5:**
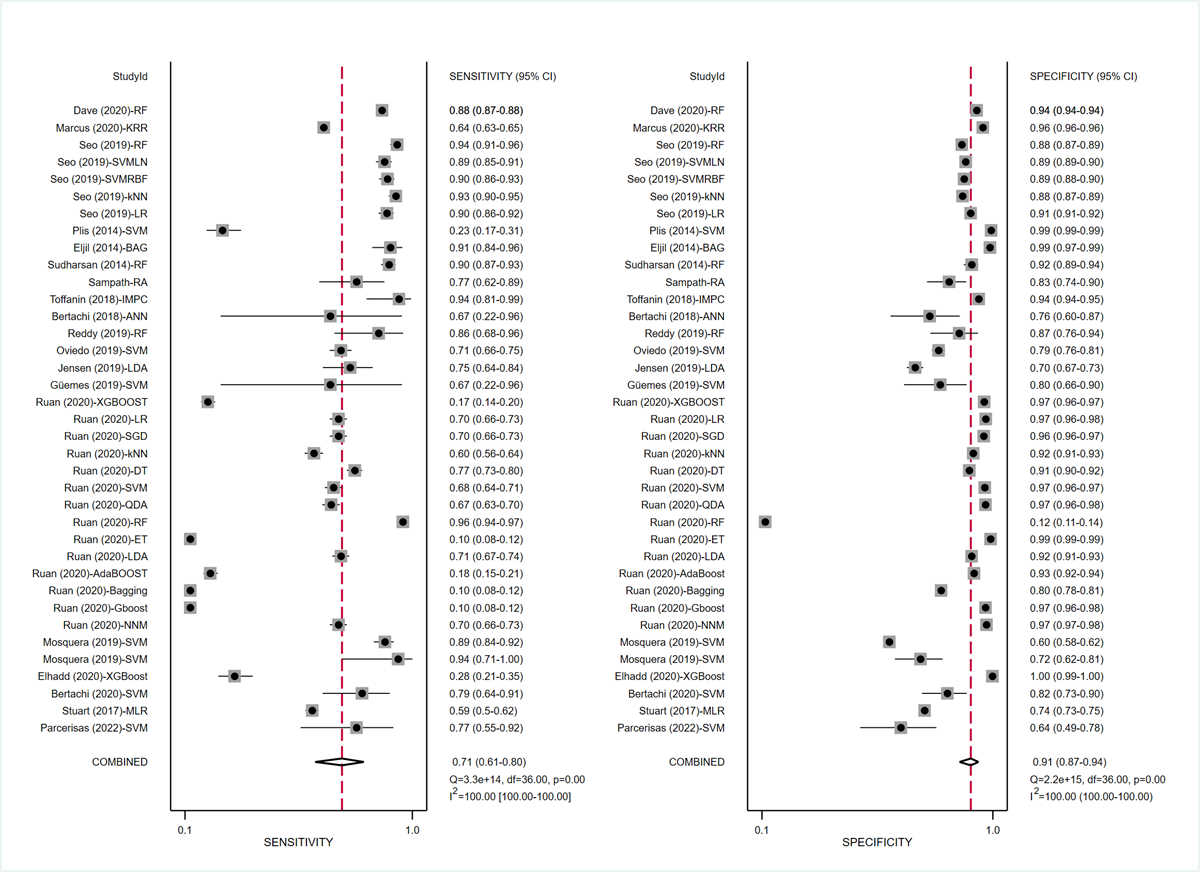
Sensitivity and specificity forest plots of ML models for predicting adverse BG events. The horizontal lines indicate 95% CIs. The square markers represent the effect value of a single study, and the diamond marker represents the combined results of all studies. The vertical line shows the line of no effects. BG: blood glucose; ML: machine learning.

**Figure 6:**
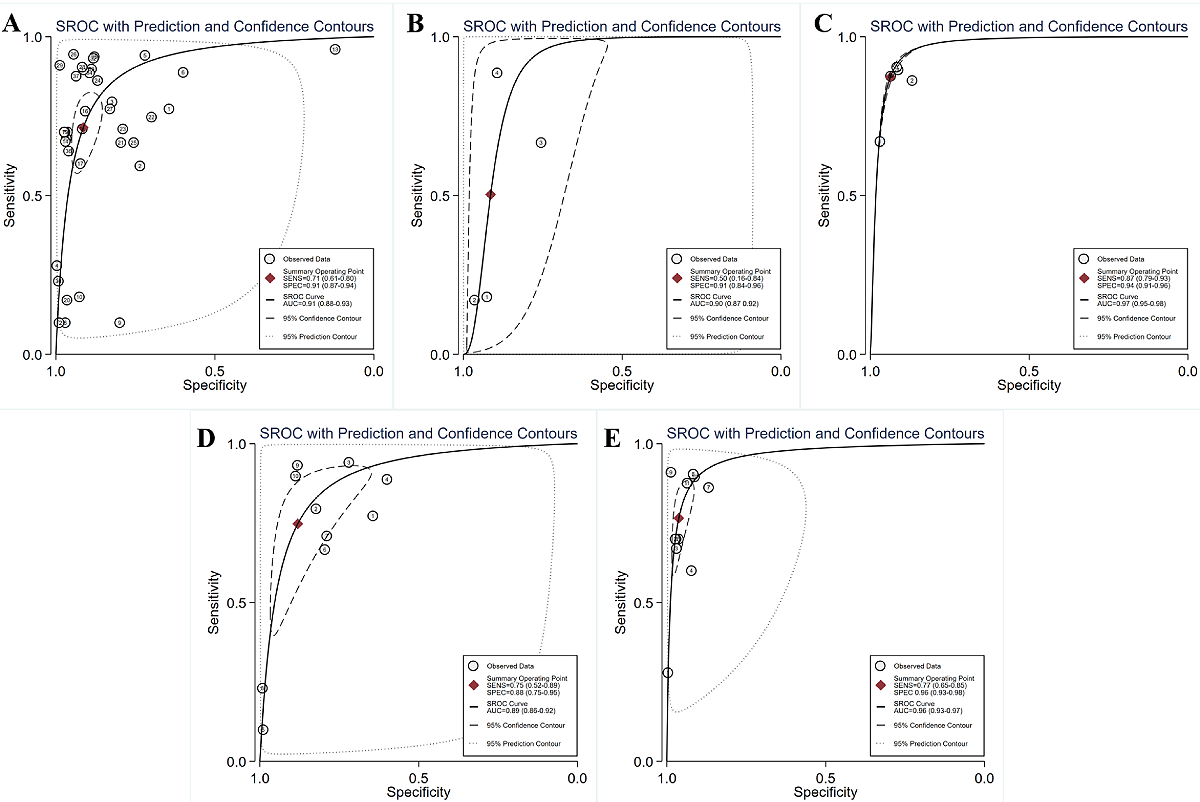
SROC curves of all ML algorithms (A), NNM algorithms (B), RF algorithms (C), SVM algorithms (D), and ensemble learning algorithms (E) for predicting adverse BG events. The hollow circles represent results of all studies, and the red diamonds represent the summary result of all studies. AUC: area under the curve; BG: blood glucose; ML: machine learning; NNM: neural network model; RF: random forest; SROC: summary receiver operating characteristic; SVM: support vector machine.

ML models for predicting hypoglycemia (adverse BG events) involved 19 (41.3%) studies [15,29-39,47,48,56-60], with pooled estimates of 0.71 (95% CI 0.61-0.80) for sensitivity, 0.91 (95% CI 0.87-0.94) for specificity, 8.3 (95% CI 5.7-12.0) for the PLR, and 0.31 (95% CI 0.22-0.44) for the NLR. The heterogeneity between different ML models in these studies is shown in the forest plot in Figure 5, which was high for both sensitivity (I²=100%, 95% CI 100%-100%) and specificity (I²=100%, 95% CI 100%-100%). The SROC curve is shown in Figure 6A, with an area under the curve (AUC) of 0.91 (95% CI 0.88-0.93). According to the meta-regression results, the type of DM and time were statistically significant sources of heterogeneity for sensitivity while the type of DM, reference, data source, setting, and threshold were statistically significant sources of heterogeneity for specificity (Multimedia Appendix 6). No statistically significant publication bias was detected (*P*=.09). In addition to integral analysis for the hypoglycemia prediction model, we also carried out analysis of 4 subgroups based on the characteristics of the included studies, including the NNM, the RF, the SVM, and ensemble learning (RF, Extreme Gradient Boosting [XGBoost], bagging).

**Figure 7:**
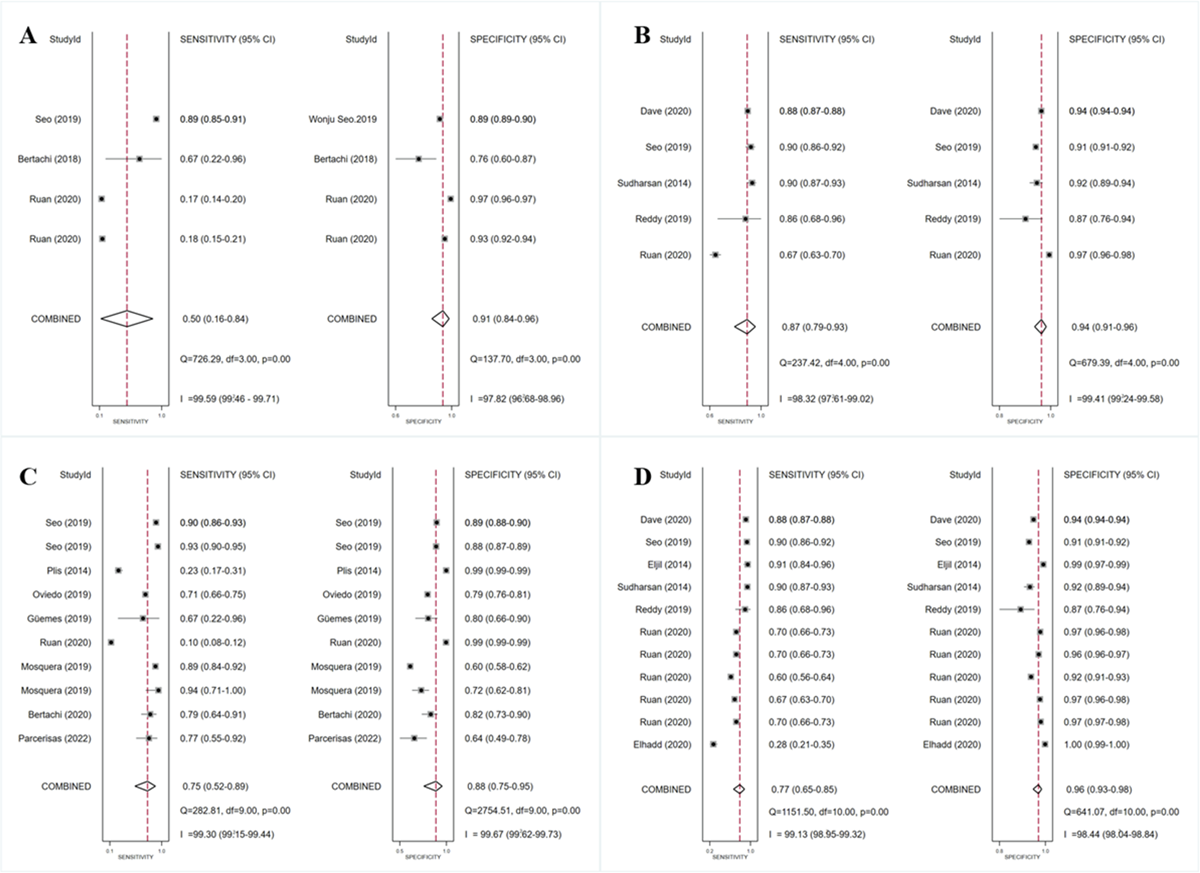
Sensitivity and specificity forest plots of NNM algorithms (A), RF models (B), SVM algorithms (C), and ensemble learning algorithms (D) for predicting adverse BG events. The horizontal lines indicate 95% CIs. The square markers represent the effect value of a single study, and the diamond marker represents the combined results of all studies. The vertical line shows the line of no effects. BG: blood glucose; NNM: neural network model; RF: random forest; SROC: summary receiver operating characteristic; SVM: support vector machine.

For the NNM, 3 (6.5%) studies [15,34,47] were included, with pooled estimates of 0.50 (95% CI 0.16-0.84) for sensitivity, 0.91 (95% CI 0.84-0.96) for specificity, 5.9 (95% CI 3.2-10.8) for the PLR, and 0.54 (95% CI 0.24-1.21) for the NLR. As shown in the forest plot in Figure 7A, I² values were 99.59% (95% CI 99.46%-99.71%) and 97.82% (95% CI 96.68%-98.86%) for sensitivity and specificity, respectively. The SROC curve is shown in Figure 6B, with an AUC of 0.90 (95% CI 0.87-0.92). Meta-regression results revealed that statistically significant heterogeneity was detected in all the factors between these studies (type of DM, reference, time, data source, setting, threshold) for sensitivity and 4 factors (reference, data source, setting, threshold) for specificity (Multimedia Appendix 7). No statistically significant publication bias was detected (*P*=.86).

For the RF, 5 (10.9%) studies [15,34,56,58,60] were included, with pooled estimates of 0.87 (95% CI 0.79-0.93) for sensitivity, 0.94 (95% CI 0.91-0.96) for specificity, 13.9 (95% CI 10.1-18.9) for the PLR, and 0.14 (95% CI 0.08-0.22) for the NLR. The forest plot in Figure 7B shows that statistically significant heterogeneity was detected in both sensitivity (I²=98.32%, 95% CI 97.61%-99.02%) and specificity (I²=99.41%, 95% CI 99.24%-99.58%). The SROC curve is shown in Figure 6C, with an AUC of 0.97 (95% CI 0.95-0.98). Meta-regression failed to run due to data instability or asymmetry. No statistically significant publication bias was detected (*P*=.21).

For the SVM, 8 (17.4%) studies [15,29,33-35,37,39,47] were involved, with pooled estimates of 0.75 (95% CI 0.52-0.89) for sensitivity, 0.88 (95% CI 0.75-0.95) for specificity, 6.3 (95% CI 3.4-11.7) for the PLR, and 0.29 (95% CI 0.15-0.55) for the NLR. Statistically significant heterogeneity was detected for both sensitivity (I²=99.30%, 95% CI 99.15%-99.44%) and specificity (I²=99.67%, 95% CI 99.62%-99.73%), as shown in Figure 7C. The SROC curve is shown in Figure 6D, with an AUC of 0.89 (95% CI 0.86-0.92). Meta-regression results showed that reference, time, data source, setting, and threshold were sources of heterogeneity for sensitivity, while reference, data source, setting, and threshold were sources of heterogeneity for specificity (Multimedia Appendix 8). Publication bias was not statistically significant (*P*=.83).

For ensemble learning models (RF, XGBoost, bagging), 7 (15.2%) studies [15,32,34,48,56,58,60] were involved, with pooled estimates of 0.77 (95% CI 0.65-0.85) for sensitivity, 0.96 (95% CI 0.93-0.98) for specificity, 20.4 (95% CI 12.5-33.3) for the PLR, and 0.24 (95% CI 0.16-0.37) for the NLR. Statistically significant heterogeneity was detected for both sensitivity (I²=99.13%, 95% CI 98.95%-99.32%) and specificity (I²=98.44%, 95% CI 98.04%-98.84%), as shown in Figure 7D. The SROC curve is shown in Figure 6E, with an AUC of 0.96 (95% CI 0.93-0.97). Meta-regression results showed that there was no source of heterogeneity for sensitivity, while the type of DM, setting, and threshold were sources of heterogeneity for specificity (Multimedia Appendix 9). No statistically significant publication bias was detected (*P*=.50).

#### Machine Learning Models for Detecting Hypoglycemia

**Figure 8:**
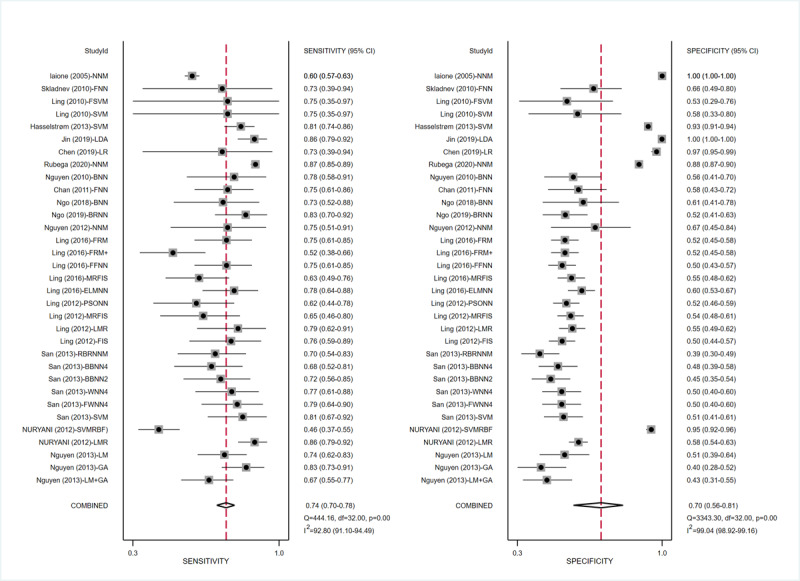
Sensitivity and specificity forest plots of ML models for detecting adverse BG events. The horizontal lines indicate 95% CIs. The square markers represent the effect value of a single study, and the diamond marker represents the combined results of all studies. The vertical line shows the line of no effects. BG: blood glucose; ML: machine learning.

**Figure 9:**
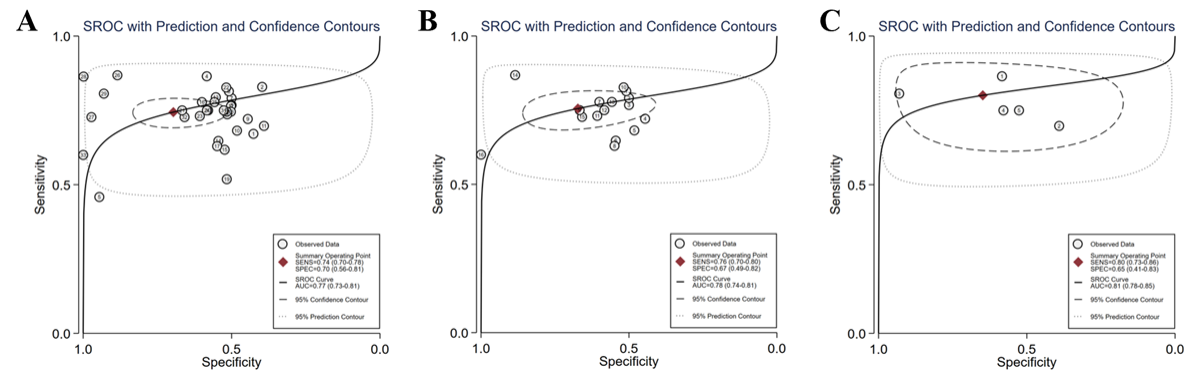
SROC curves of all ML algorithms (A), NNM algorithms (B), and SVM algorithms (C) for detecting adverse BG events. The hollow circles represent results of all studies, and the red diamonds represent the summary result of all studies. AUC: area under the curve; BG: blood glucose; ML: machine learning; NNM: neural network model; SROC: summary receiver operating characteristic; SVM: support vector machine.

ML models for detecting hypoglycemia (adverse BG events) involved 17 (37%) studies [10,16,40-46,61-68], with pooled estimates of 0.74 (95% CI 0.70-0.78) for sensitivity, 0.70 (95% CI 0.56-0.81) for specificity, 2.4 (95% CI 1.6-3.7) for the PLR, and 0.37 (95% CI 0.29-0.46) for the NLR. The heterogeneity between different models in these studies is shown in the forest plots in Figure 8 and was high for both sensitivity (I²=92.80%, 95% CI 91.10%-94.49%) and specificity (I²=99.04%, 95% CI 98.82%-99.16%). The SROC curve is shown in Figure 9A, with an AUC of 0.77 (95% CI 0.73-0.81). Based on the meta-regression results, reference, time, data source, setting, and threshold were statistically significant sources of heterogeneity for sensitivity, while reference, data source, and threshold were statistically significant sources of heterogeneity for specificity (Multimedia Appendix 9). Statistically significant publication bias was detected (*P*<.001). In addition to integral analysis for the hypoglycemia detection model, we also carried out analysis of 2 subgroups based on the characteristics of the included studies, including the NNM and the SVM.

**Figure 10:**
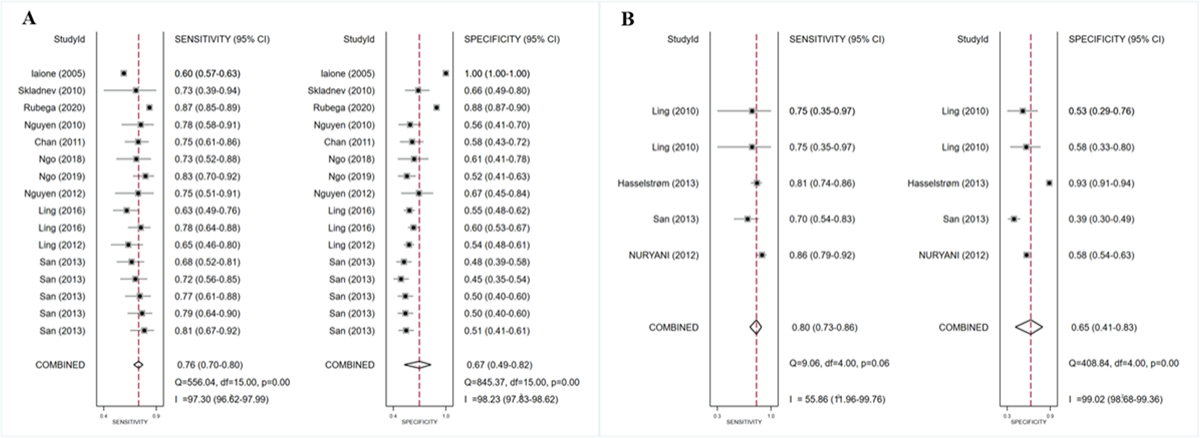
Sensitivity and specificity forest plots of NNM algorithms (A) and SVM algorithms (B) for detecting adverse BG events. The horizontal lines indicate 95% CIs. The square markers represent the effect value of a single study, and the diamond marker represents the combined results of all studies. The vertical line shows the line of no effects. BG: blood glucose; NNM: neural network model; SVM: support vector machine.

For the NNM, 11 (23.9%) studies [40-42,45,46,62-67] were involved, with pooled estimates of 0.76 (95% CI 0.70-0.80) for sensitivity, 0.67 (95% CI 0.49-0.82) for specificity, 2.3 (95% CI 1.4-3.9) for the PLR, and 0.36 (95% CI 0.27-0.48) for the NLR. The heterogeneity between different studies is shown in the forest plot in Figure 10A and was high for both sensitivity (I²=97.30%, 95% CI 96.62%-97.99%) and specificity (I²=98.23%, 95% CI 97.83%-98.62%). The SROC curve is shown in Figure 9B, with an AUC of 0.78 (95% CI 0.74-0.81). Based on the of meta-regression results, reference, time, data source, setting, and threshold were statistically significant sources of heterogeneity for sensitivity, while reference and setting were statistically significant sources of heterogeneity for specificity (Multimedia Appendix 10). Statistically significant publication bias was detected (*P*<.001).

For the SVM, 4 (8.7%) studies [10,44,61,62] were included, with pooled estimates of 0.80 (95% CI 0.73-0.86) for sensitivity, 0.65 (95% CI 0.41-0.83) for specificity, 2.3 (95% CI 1.2-4.4) for the PLR, and 0.31 (95% CI 0.18-0.51) for the NLR. The heterogeneity between different studies is shown in the forest plot in Figure 10B and was high for both sensitivity (I²=55.86%, 95% CI 11.96%-99.76%) and specificity (I²=99.02%, 95% CI 98.68%-99.36%). The SROC curve is shown in Figure 9C, with an AUC of 0.81 (95% CI 0.78-0.85). Meta-regression results indicated that reference, time, data source, setting, and threshold were statistically significant sources of heterogeneity for sensitivity, while reference, data source, setting, and threshold statistically significant sources of heterogeneity for specificity (Multimedia Appendix 11). No statistically significant publication bias was detected (*P*=.31).

## Discussion

### Principal Findings

This meta-analysis systematically assessed the performance of different ML models in enhancing BG management in patients with DM based on 46 eligible studies. Comprehensive evidence obtained via exhaustive searching allowed us to assess the overall ability of the ML models in different scenarios, including predicting BG levels, predicting adverse BG events, and detecting adverse BG events.

### Comparison to Prior Work

Obviously, the RMSE of ML models for predicting BG levels increased as the PH increased from 15 to 60 minutes, which indicates that the longer the PH, the larger the prediction error. Based on the results of relative ranking, among all the ML models for predicting BG levels, neural network–based models, including the DRNN, GluNet, ARJNN, and NNM, achieved the minimum RMSE and the maximum SUCRA in different PHs, indicting the highest relative performance. In contrast, the DT achieved the maximum RMSE and the minimum SUCRA in a PH of 60 and 45 minutes, indicating that lowest relative performance. Thus, for predicting BG levels, neural network–based algorithms might be an appropriate choice. We found that time domain features combined with historical BG levels as input can further improve the performance of NNM algorithms [49,55]. However, the quality of training data for NNMs needs to be high; therefore, the requirements during data collection and preprocessing of raw data are high [22,51].

Regarding ML models for predicting adverse BG events, the pooled sensitivity, specificity, PLR, and NLR were 0.71 (95% CI 0.61-0.80), 0.91 (95% CI 0.87-0.94), 8.3 (95% CI 5.7-12.0), and 0.31 (95% CI 0.22-0.44), respectively. According to the *Users’ Guide to Medical Literature*, with regard to diagnostic tests [69], a PLR of 5-10 should be able to moderately increase the probability of persons having or developing a disease and an NLR of 0.1-0.2 should be able to moderately decrease the probability of having or developing a disease after taking the index test. Hence, current ML models have relatively sufficient ability to predict the occurrence of hypoglycemia, especially RF algorithms with a PLR of 13.9 (95% CI 10.1-18.9) and an NLR of 0.14 (95% CI 0.08-0.22). On the contrary, although the PLR of NNM algorithms was 5.9 (95% CI 3.2-10.8), their sensitivity and NLR were 0.50 (95% CI 0.16-0.84) and 0.54 (95% CI 0.24-1.21), respectively, which is far from satisfactory. Although RF algorithms seem to be able to capture the complex, nonlinear patterns affecting hypoglycemia [56], it was still not enough to determine which algorithm shows the best performance, as the test scenarios were quite different and there was high heterogeneity between studies.

Regarding ML models for detecting hypoglycemia, the pooled sensitivity, specificity, PLR, and NLR were 0.74 (95% CI 0.70-0.78), 0.70 (0.56-0.81), 2.4 (1.6-3.7), and 0.37 (0.29-0.46), respectively, which indicates that the algorithms generate small changes in probability [69]. Nevertheless, it does not mean that ML models combined with ECG or EEG monitoring, which we found in 13 of 17 studies, should not be further investigated. Considering patients with both DM and cardiovascular risk, or patients under intensive care and in a coma, combined ML models and ECG or EEG signals might be able to avoid deficits in physical and cognitive function and death caused by hypoglycemia [70].

### Strengths and Limitations

The study has several limitations. First, although we developed a comprehensive search strategy, there was still a possibility of potential missing studies. To further increase the rate of literature retrieval, we included the main medical databases with a feasible search strategy, including PubMed, Embase, Web of Science, and IEEE Explore, and references from relevant studies were also screened for eligibility to avoid omissions. Second, statistically significant high heterogeneity was detected in all subgroups, with different sources of heterogeneity, including different types of DM, ML models, data sources, reference index, time and setting of data collection, and threshold of hypoglycemia, among studies. To address this issue, hierarchical analysis and meta-regression analysis were carried out in different subgroups to explore the possible sources of heterogeneity. Furthermore, for several studies that provided no required outcome measures or had inconsistent outcome measures, relevant estimation methods were used to calculate the indicators, which might have led to a certain amount of estimation error. However, the estimation error was small enough to be accepted owing to an appropriate estimation method, and the results of this study were further enriched. However, future studies are required to report all relevant outcome measures for further evaluation.

### Future Directions

In future, more accurate ML models will be used for BG management, which will certainly improve the quality of life of patients with DM and reduce the burden of adverse BG events. First, as mentioned before, current ML models have relatively sufficient ability to predict BG levels and hypoglycemia, and the fact that an extended PH is more beneficial for increasing the time available for patients and clinicians to respond still needs to be emphasized [15]. Hence, future studies should focus on enhancing the performance of ML models in longer PHs (ie, 60 minutes). Second, most of the raw data from CGM devices are highly imbalanced due to the low incidence of adverse BG events, which may lead to several performance distortions. Previous studies have reported several approaches to reduce the data imbalance, including oversampling [71] and cost-based learning [15]. However, to the best of our knowledge, few studies have investigated the effectiveness of those approaches in BG management models, which needs to be further studied in the future. Furthermore, the high variability of BG levels in the human body due to several factors, such as meal intake, high-intensity exercise, and insulin dosage, creates challenges for ML models; thus, future works need to integrate these factors with existing models to further enhance their accuracy [22,51]. It is also necessary to consider the computational complexity and convenience of use for patients and physicians. Moreover, several studies have implied that a combination of ML models and features extracted from CGM profiles can achieve better predictability compared to an ML model alone [15,56]. Recently, studies have focused on more novel deep learning models, such as transformers, which have also been proved clinically useful [72]. Therefore, further studies that focus on optimizing the structure of an ensemble method are needed to explore more models with a new structure. Lastly, it should be mentioned that although several studies have achieved high performance using relatively small data set [29,31,32,35,39,47,57], which can reduce the difficulty in model development, it also creates a concern about whether this will decrease the generalization ability of the models. Most of the models were developed and tested with a certain data set, and few of them have been prospectively validated in a clinical setting. Therefore, they need to be applied in clinical practice and be updated, as needed, to provide real-time feedback for the automatic collection of BG levels and generate a basis for prompt medical intervention [73].

### Conclusion

In summary, in predicting precise BG levels, the RMSE increases with an increase in the PH, and the NNM shows the relatively highest performance among all the ML models. Meanwhile, according to the PLR and NLR, current ML models have sufficient ability to predict adverse BG (hypoglycemia) events, while their ability to detect adverse BG events needs to be enhanced. Future studies are required to focus on improving the performance and using ML models in clinical practice [70,73].

## Appendix

Multimedia Appendix 1 Supplemental plot1-forest (RMSE PH=30). PH: prediction horizon; RMSE: root mean square error.

Multimedia Appendix 2 PRISMA (Preferred Reporting Items for Systematic Reviews and Meta-Analysis) checklist.

Multimedia Appendix 3 Supplemental plot2-forest (RMSE PH=60). PH: prediction horizon; RMSE: root mean square error.

Multimedia Appendix 4 Supplemental plot3-forest (RMSE PH=15). PH: prediction horizon; RMSE: root mean square error.

Multimedia Appendix 5 Supplemental plot4-forest (RMSE PH=45). PH: prediction horizon; RMSE: root mean square error.

Multimedia Appendix 6 Supplemental plot5 - metaregression (pre-all).

Multimedia Appendix 7 Supplemental plot5-metaregression(pre-NN).

Multimedia Appendix 8 Supplemental plot5-metaregression(pre-SVM).

Multimedia Appendix 9 Supplemental plot5-metaregression(det-all).

Multimedia Appendix 10 supplemental plot5-metaregression(det-NN).

Multimedia Appendix 11 Supplemental plot5-metaregression(det-SVM).

## Abbreviations

ARM: autoregression model
ARJNN: ARTiDe jump neural network
AUC: area under the curve
BG: blood glucose
CGM: continuous glucose monitoring
DM: diabetes mellitus
DRNN: dilated recurrent neural network
DT: decision tree
ECG: electrocardiograph
EEG: electroencephalograph
EHR: electronic health record
ML: machine learning
NLR: negative likelihood ratio
NNM: neural network model
PH: prediction horizon
PLR: positive likelihood ratio
QUADAS-2: Quality Assessment of Diagnostic Accuracy Studies
RF: random forest
RMSE: root mean square error
SROC: summary receiver operating characteristic
SUCRA: surface under the cumulative ranking
SVM: support vector machine
T1DM: type 1 diabetes mellitus
T2DM: type 2 diabetes mellitus
XGBoost: Extreme Gradient Boosting

## Data Availability

The data sets used and analyzed during the study are available from the corresponding author upon reasonable request.
