# Supplementary figures and images for: Machine Learning Models for Blood Glucose Level Prediction in Patients With Diabetes Mellitus: Systematic Review and Network Meta-Analysis
Source: JMIR Med Inform. 2023 Nov 20;11:e47833. doi: 10.2196/47833 (PMC10696506; doi:10.2196/47833)

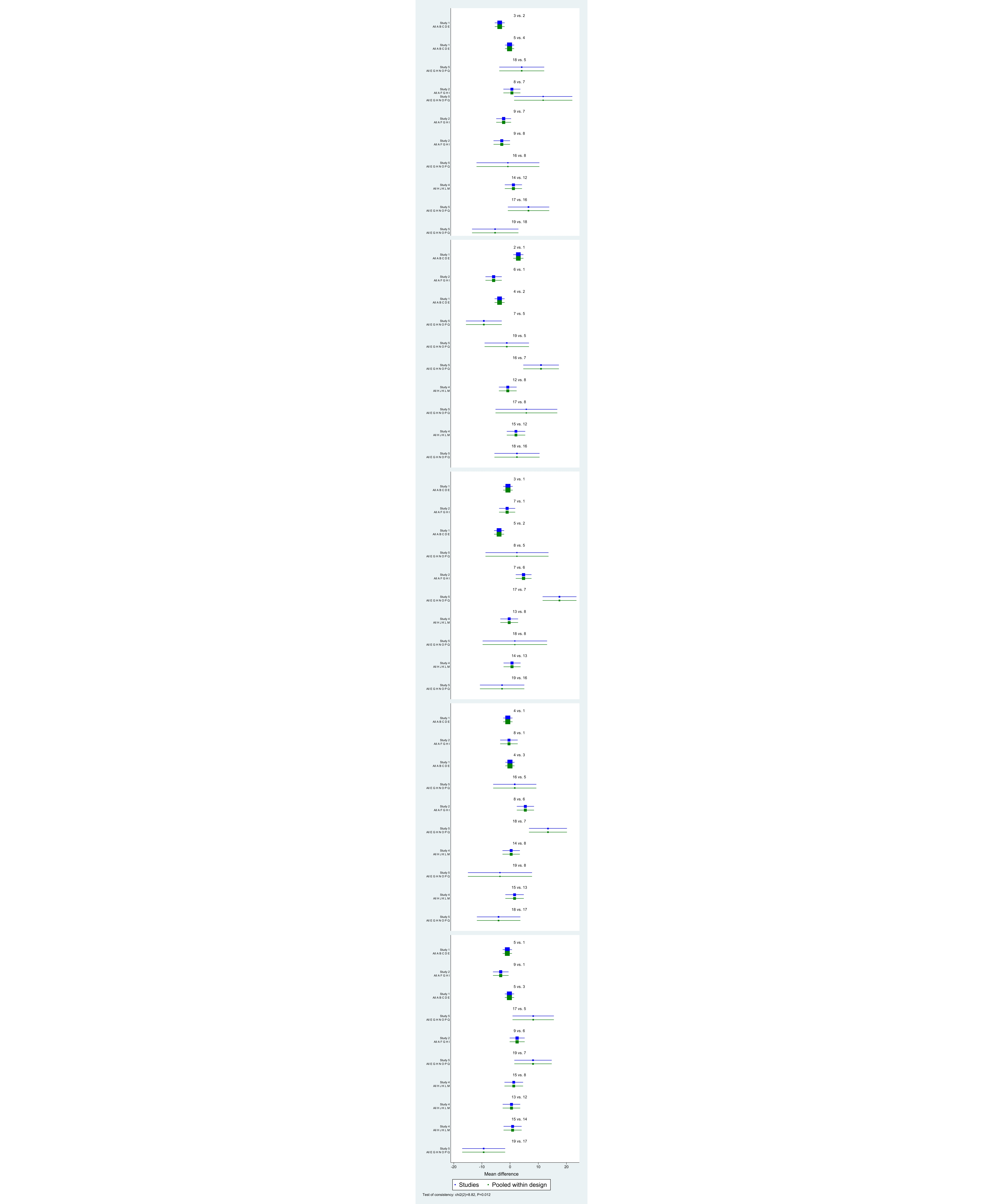

Supplement: Multimedia Appendix 3 [file medinform_v11i1e47833_app3.png]

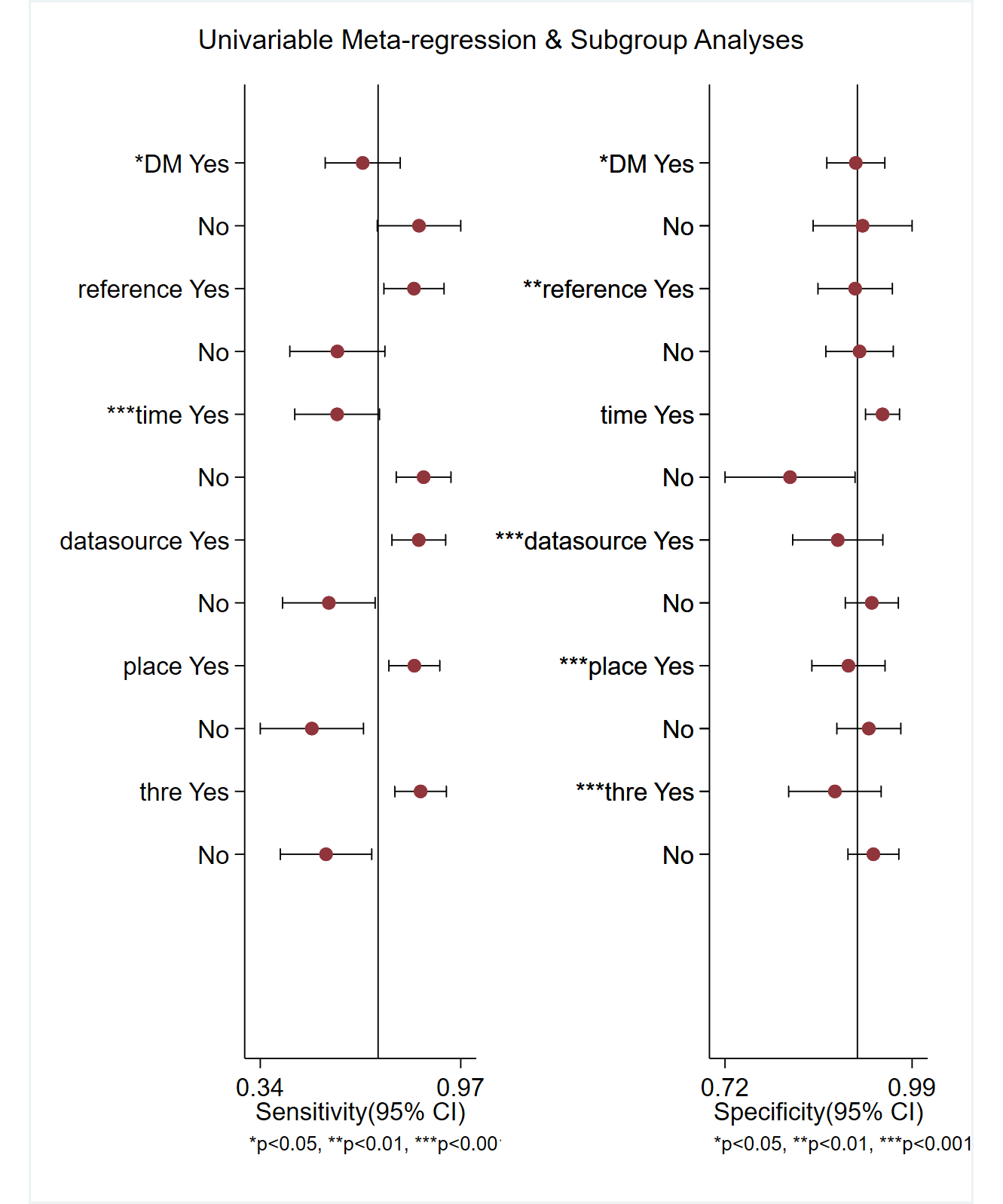

Supplement: Multimedia Appendix 6 [file medinform_v11i1e47833_app6.png]

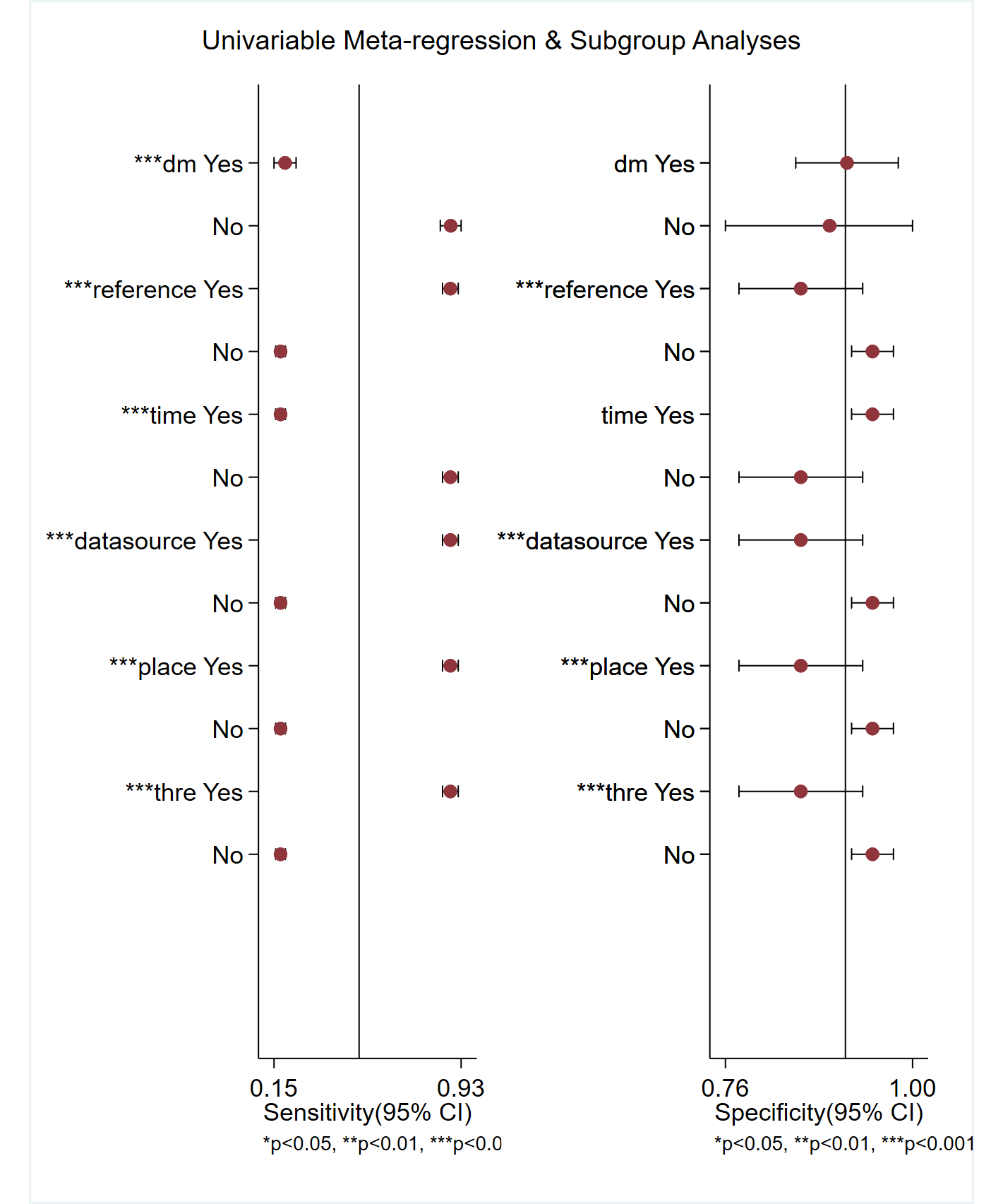

Supplement: Multimedia Appendix 7 [file medinform_v11i1e47833_app7.png]

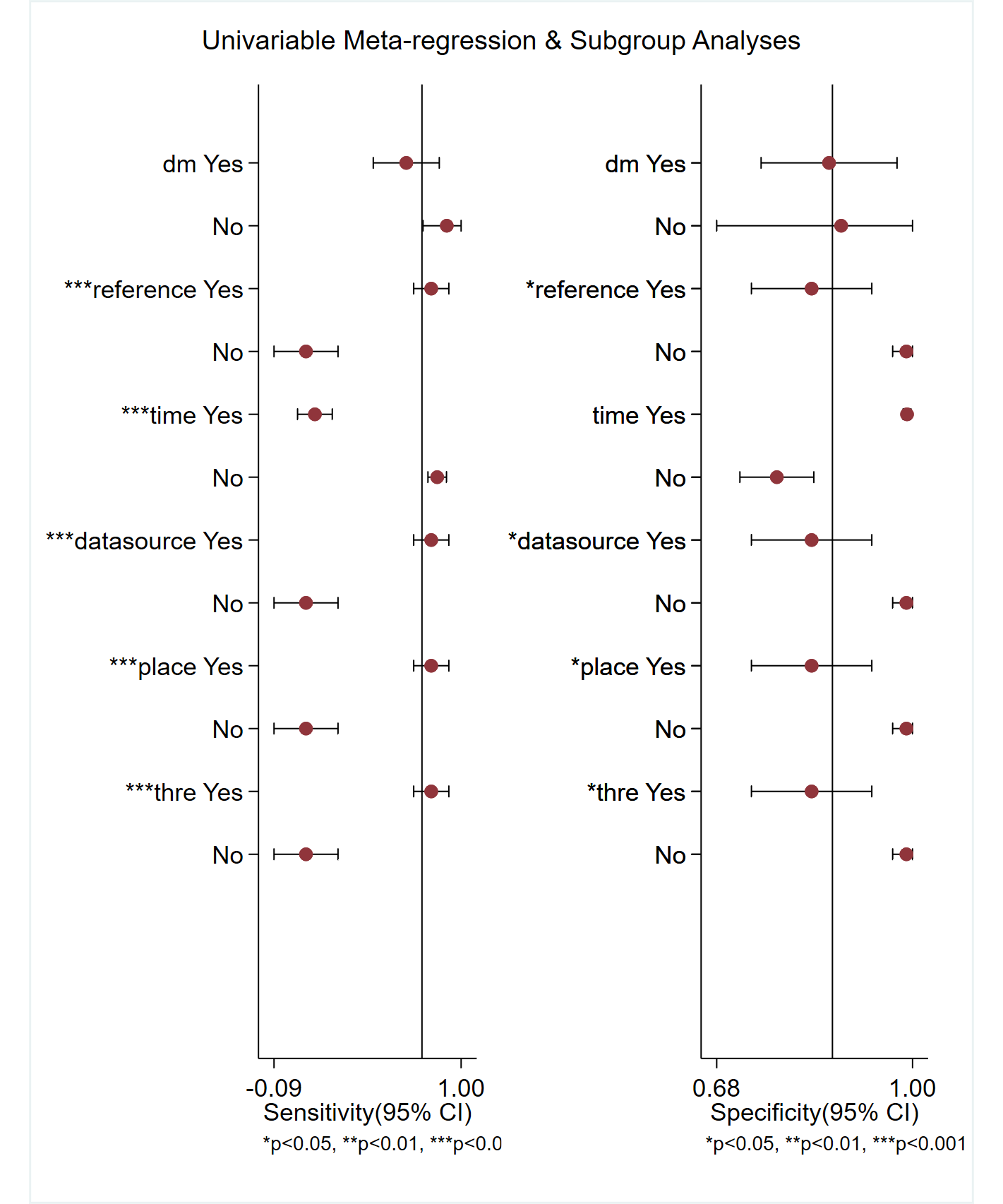

Supplement: Multimedia Appendix 8 [file medinform_v11i1e47833_app8.png]

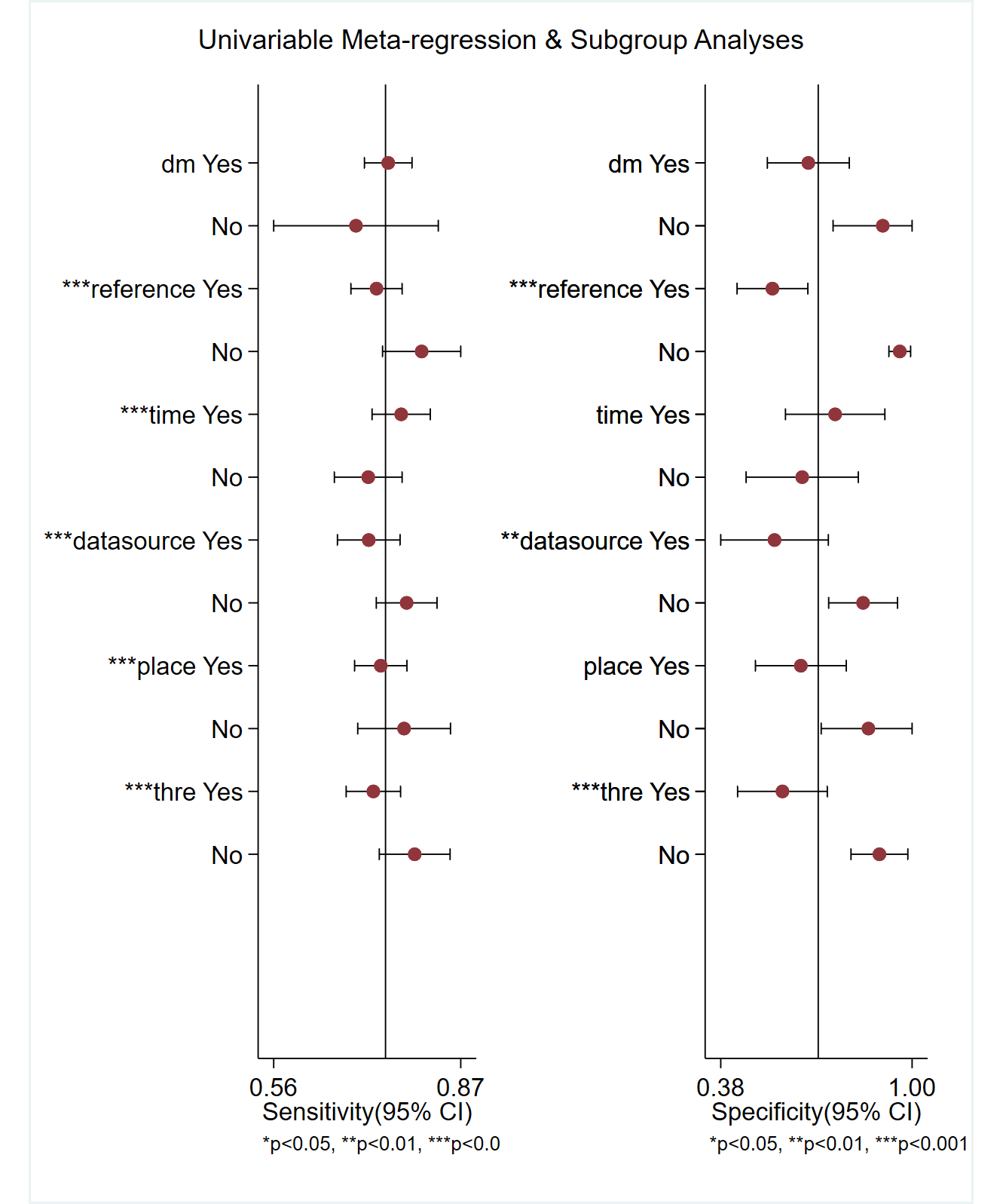

Supplement: Multimedia Appendix 9 [file medinform_v11i1e47833_app9.png]

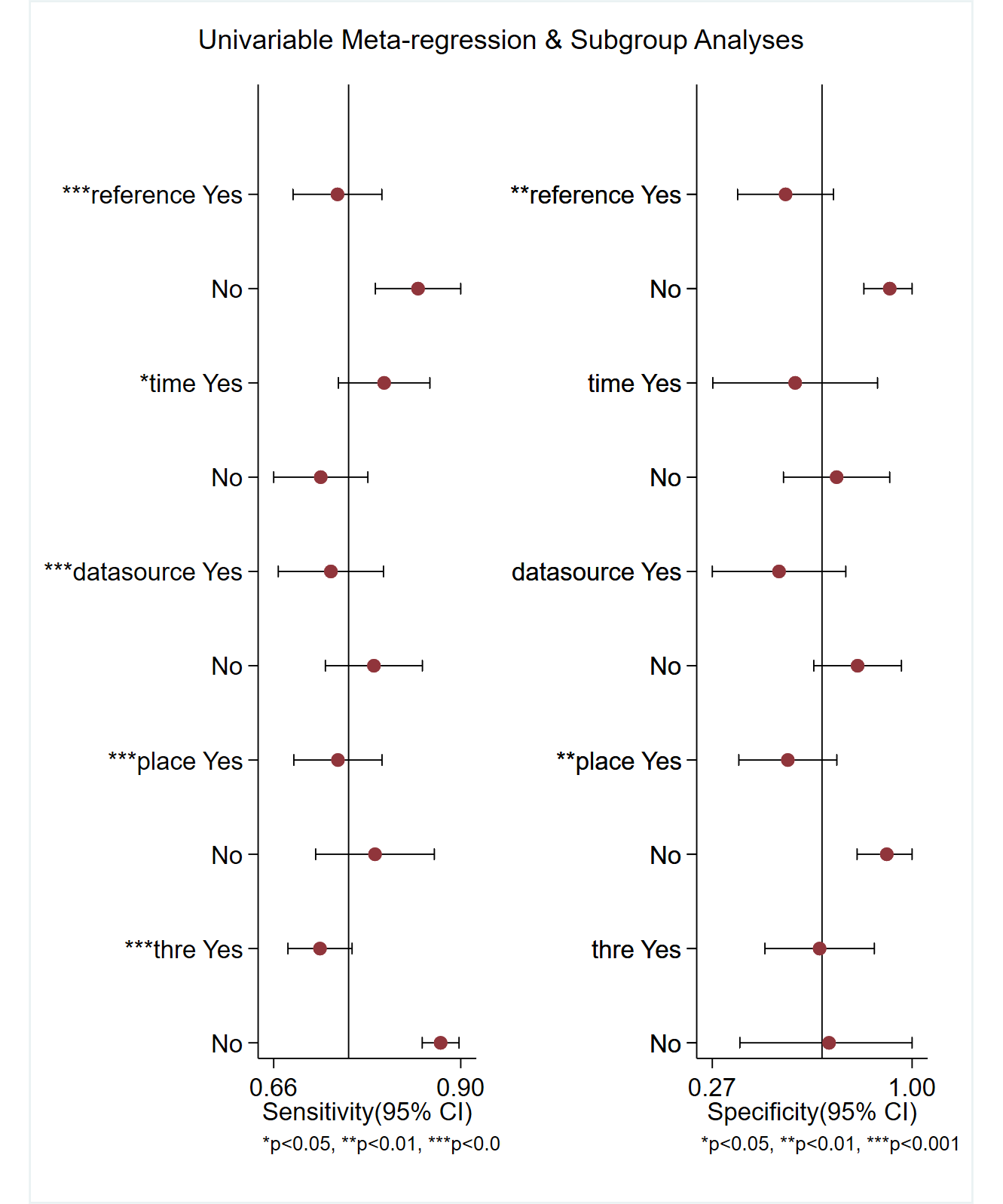

Supplement: Multimedia Appendix 10 [file medinform_v11i1e47833_app10.png]

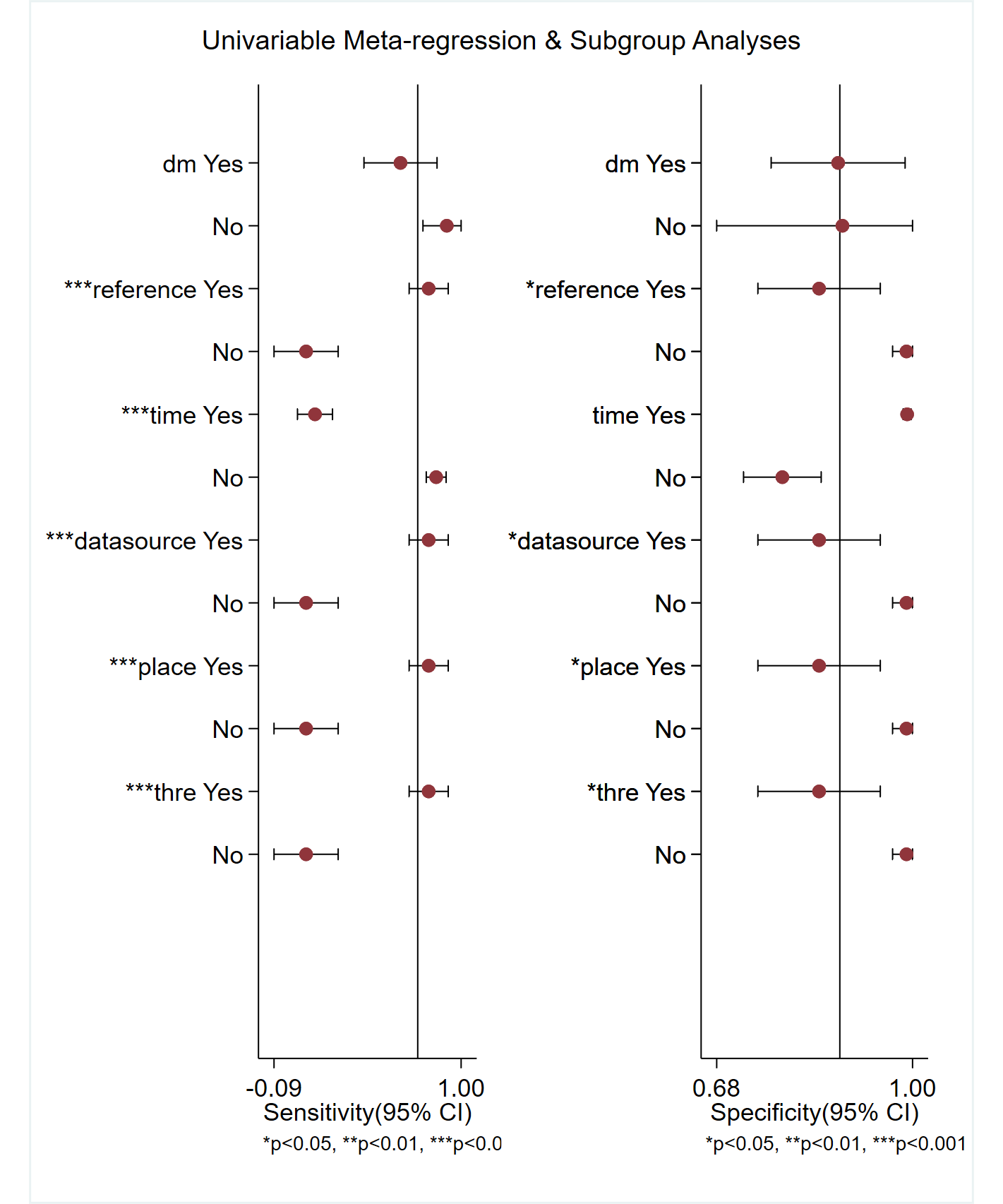

Supplement: Multimedia Appendix 11 [file medinform_v11i1e47833_app11.png]
